# Non-coding RNA therapeutics in cardiovascular diseases and risk factors: Systematic review

**DOI:** 10.1016/j.ncrna.2023.06.002

**Published:** 2023-07-01

**Authors:** Meity Ardiana, Asiyah Nurul Fadila, Zakirah Zuhra, Nabilla Merdika Kusuma, Made Edgard Surya Erlangga Rurus, Delvac Oceandy

**Affiliations:** a Department of Cardiology and Vascular Medicine, Dr.Soetomo General Hospital, Surabaya, Indonesia; b Faculty of Medicine, Airlangga University, Surabaya, Indonesia; c Faculty of Medicine, University of Indonesia, Jakarta, Indonesia; d Faculty of Medicine, Hang Tuah University, Surabaya, Indonesia; e Division of Cardiovascular Sciences, Faculty of Biology, Medicine and Health, The University of Manchester, Manchester, United Kingdom

**Keywords:** Non-coding RNA, RNA therapeutics, Cardiovascular diseases, Cardiovascular risk factors, Cardiogenetics

## Abstract

At present, RNA-based therapy which includes therapies using non-coding RNAs (ncRNAs), antisense oligonucleotides (ASOs), and aptamers are gaining widespread attention as possible ways to target genes in various cardiovascular diseases (CVDs), thereby serving as a promising therapeutic approach for CVDs and risk factors management. However, data are primarily in an early stage. A systematic review was carried out using literature from several databases (Pubmed, Cochrane, Scopus, and DOAJR) following the PRISMA guidelines. Of the 64 articles reviewed, 39 papers were included in this review with three main types of RNAs: aptamers, antisense oligonucleotides (ASOs), and small-interfering RNA (siRNA). All studies were human clinical trials. RNA-based therapies were demonstrated to be efficacious in treating various CVDs and controlling cardiovascular risk factors. They are generally safe and well-tolerated. However, data are still in the early stage and warrant further investigation.

## 1 Introduction

Despite the current advance in therapeutic strategies, cardiovascular diseases (CVDs) are still a leading cause of mortality worldwide, and CV risk factors such as hyperlipidemia, diabetes, and hypertension further contribute to the progression of CVDs [1]. RNA-based therapeutics have emerged as a vastly growing field that holds promising value in diagnosing and managing diverse health conditions, including CVDs and management of CV risk factors. Different types of RNA have been known to play a crucial role in regulating gene expression that is responsible for health outcomes in many CVDs [2,3]. Exogenous RNA agents offer varying mechanisms of action, including gene silencing, mRNA editing, or replacement, which further broaden the possibility of addressing different pathophysiological pathways in varying diseases. Thus, this novel therapy may aid in the treatment and prevention of CVDs along with managing its risk factors [4].

Several strategies have been developed to target gene expression including the use of antisense oligonucleotides (ASO), aptamers, small-interfering RNA (siRNA), microRNA (miRNA), and messenger RNA (mRNA) [2,3]. siRNA and miRNA target specific endogenous mRNA to inhibit the subsequent protein translation. In contrast, ASOs bind to mRNA to block its function [5]. A newly-invented agent, aptamers, also binds to specific target molecules through a repetitive process termed *systemic evolutions of ligands by exponential enrichment* (SELEX) [6]. Meanwhile, mRNA therapy utilizes modified mRNA to directly encode functional proteins to cure certain conditions. This approach demonstrated excellent therapeutic results in several different CVDs, such as atrial fibrillation and coronary artery diseases [7]. Furthermore, the use of RNA-based therapy also manages risk factors in CVD, such as hyperlipidemia and diabetes mellitus [8,9].

Despite all this exciting progress, many caveats still need to be addressed before widely implementing RNA-based therapy in clinical settings. This study aims to evaluate and summarize current advances in the field of RNA-based therapy for the treatment of CVDs and provide a comprehensive overview regarding the use of non-coding RNA therapeutics in managing CVDs and their risk factors.

## 2 Material and methods

### 2.1 Search strategy

This systematic review was conducted following the Preferred Reporting Items for Systematic Reviews and Meta-Analyses (PRISMA) guidelines. Electronic databases were systematically searched, including Pubmed, Scopus, and the Cochrane Library. The PICO in this review was as follows; P: adult patients with CVDs and/or risk factors, I: RNA-based therapy, C: placebo/control, O: efficacy or safety of treatment. Relevant keywords and Medical Subject Headings (MeSH) terms were utilized, such as “RNA therapeutics”, “RNA therapy”, “Cardiovascular Diseases”, “Cardiovascular Risk Factors”, and “Management”. Further details of search keywords are shown in **Supplementary 1.** We also conducted a manual search using the snowballing method by checking reference lists from related studies and Google searches to avoid missing pertinent articles. Data searching was performed from January-February 2023. Study designs were randomized controlled trials (RCTs), clinical, observational, and multi-center studies. In addition, other search filters were applied, such as studies on human participants, language (English), and full article availability online. Due to the novel nature of our topic, we did not limit the time of publication of included article results.

### 2.2 Study selection criteria

**Fig. 1:**
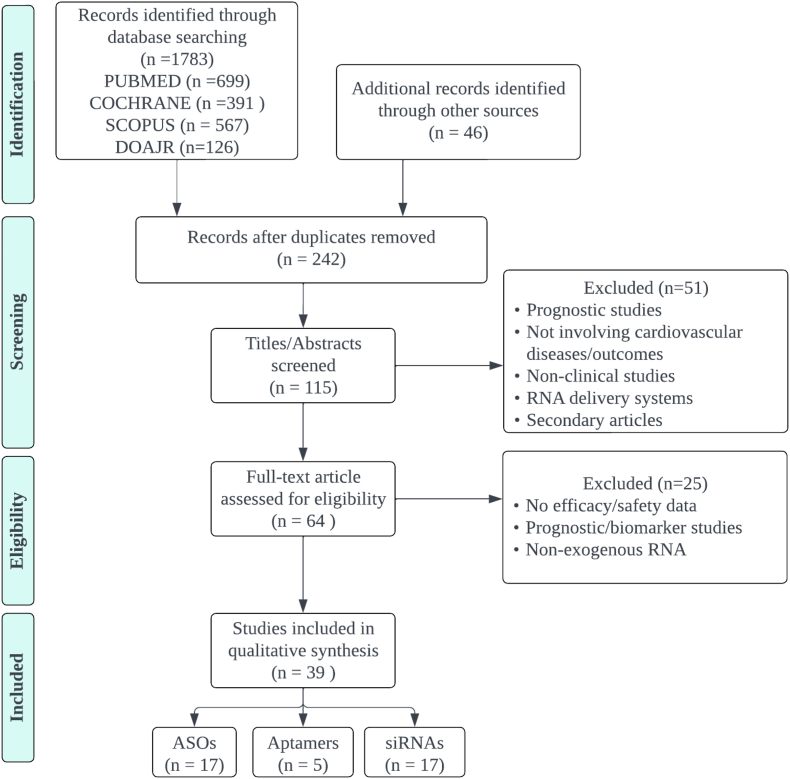
Literature search strategy PRISMA flow diagram.

The inclusion criteria used to select relevant studies for this systematic review are as follows: clinical trials; adult population (18 years and above) with a record of CVDs and/or risk factors (heart failure, myocardial disease, reperfusion injury, ischemic heart diseases, arrhythmia, dyslipidemia, hypercholesterolemia, diabetes mellitus, hypertension, peripheral artery diseases, vascular thromboembolism, amyloidosis, and cardiomyopathy); used exogenous RNA-based therapy; had a comparison/control group in the study assessing efficacy; and reported clear outcome measures for efficacy and safety. We excluded in-vitro or preclinical studies, endogenous RNA as biomarker/target therapy, pharmacokinetics and pharmacodynamics studies, route of delivery, no efficacy outcome measures reported, and secondary articles such as reviews and meta-analyses. After the initial literature search, duplicates and irrelevant materials (i.e., scientific posters, conference abstracts, editorials, and letters to the editor) were removed. Two independent reviewers (NM & ZZ) screened article titles and abstracts to assess their eligibility. Any disagreement would be resolved by group discussion with other reviewers (AN & ME) and a senior reviewer (MA). If eligibility were unclear, the article would be retrieved for further clarification. Full texts of all relevant articles were subsequently retrieved. The search process and results are shown in the PRISMA flow diagram (Fig. 1).

### 2.3 Data extraction and quality assessment

After the abstract screening, 115 articles were included. Two independent reviewers (AN & ER) read each article's protocols, full text, and supplementary materials. Data extracted were of the following: (1) Study characteristics, including first author, year of publication, study design, and patient demographics; (2) Types of RNA therapeutics used in the study, including dosing, route of administration, frequency, and duration; (3) Sample size, including controls and their treatments; (4) Primary and other relevant outcomes of the study, along with each measurement; (5) Results in the form of any significant findings, statistical data, and *p-*values, as well as any adverse events documented in the study. We reported the mean difference between treatment and control groups on primary or related outcomes.

Then, using the Cochrane risk-of-bias tool for randomized trials (RoB 2) for RCTs and the Risk Of Bias In Non-Randomized Studies - of Interventions (ROBINS-I) for non-randomized clinical trials, two reviewers independently assessed the risk of bias. RoB 2 is classified into five main domains, namely assessment of bias arising from; (1) The randomization process, (2) Deviations from intended interventions, (3) Missing the outcome data, (4) Measurement of the outcome, and (5) Selective reporting. Meanwhile, ROBINS-1 divides into seven domains assessing bias from confounding factors, selection of participants, intervention classification, missing data, measurement of outcomes, selective reporting, and other sources of bias. The assessment will be summarized in the form of a Risk-of-Bias visualization tool (Robvis).

## 3 Results and discussion

### 3.1 Exogenous RNA therapeutics in various cardiovascular diseases

#### 3.1.1 Antisense oligonucleotides (ASO)

**Fig. 2:**
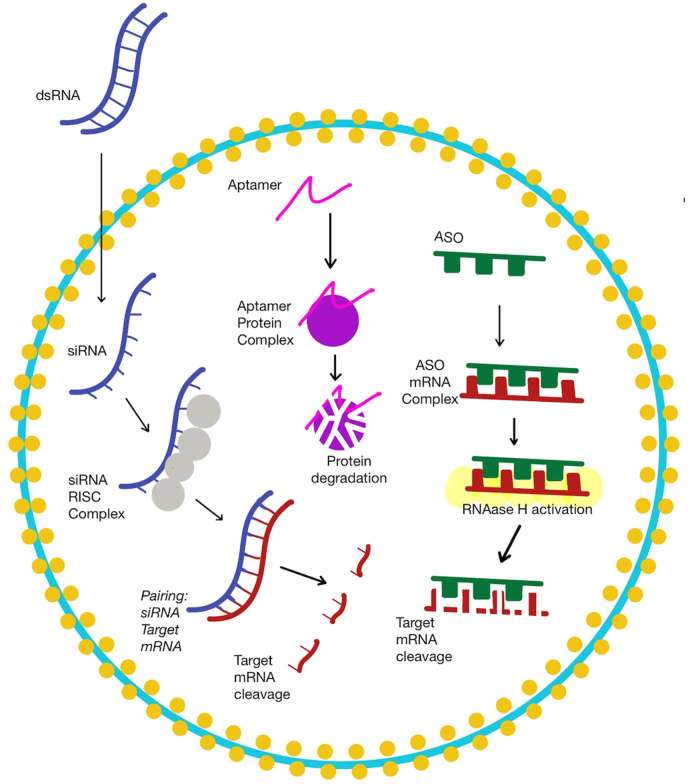
The Mechanism of Actions of ASO, Aptamer and siRNA.

Antisense oligonucleotides (ASO) is a synthetic agent made of short single-stranded DNA-based oligonucleotides binding to target mRNA to inhibit protein translation via Watson-Crick base-pairing and typically has 20 base pairs [10]. ASO targets various classes of nucleic acids, such as mRNA, pre-mRNA, and non-coding RNA. It allows the inhibition of protein production by forming a duplex with the target mRNA and stimulating RNAase H that degrades the target mRNA. RNA/ASO duplex can alternatively block pre-mRNA processing and mRNA translation [5] (Fig. 2). ASOs are subject to modifications, which may improve the pharmacodynamic and pharmacokinetic properties. It has been studied for both neurodegenerative diseases and cardiovascular diseases (CVDs) [2].

Table 1 illustrates the use of ASO as therapy in studies of CVDs and risk factors, drug monitoring, and outcomes, including death, MACE, and allergic reactions. The most studied CVDs were hypercholesterolemia (55%,11/20 studies), transthyretin amyloidosis (20%, 4/20 studies), atherosclerotic cardiovascular disease (ASCVD), and atrial fibrillation. The most studied ASOs were mipomersen (20%, 5/20 studies) and inotersen (15%, 3/20 studies).

**Table 1:** ASO.

No	Study registration number (ref)	Study Population	Age Group	Intervention Arm	n	Comparator arm	n	Time to follow Up	Primary Endpoint	Outcome: Mean Difference (Treatment vs Placebo)	Adverse Events
1	NCT00216463 (Furtado, 2012)	hypercholesterolemic LDL-C ≥130 mg/dL and TG ≤ 4oomg/dL BMI 25–32kg/m2	18–65 years old	Mipomersen once a week. Doses 100 mg 200 mg 300 mg For a total of 13 weeks	8 8 6 (apoCIΩ	Placebo	2 2 2	Day 99	Total cholesterol; Concentration of ApoCIII; concentration of apoB	Total Cholesterol 100 mg = −33.3 (−60.6, −5.9) p = 0.004 200 mg = −78.4 (−105.7, −51.1) p < 0.001 300 mg = −108.5 (−136.9, −81.0) p < 0.001 ApoB 100 mg = −30.3 (−46.2, −14.3) p = 0.001 200 mg = −57.3 (−72.3, −42.3) p < 0.001 300 mg = −84.5 (−100.3, −68.7) p < 0.001 ApoCIII 100 mg = 0.97 (−3.1, 5.1) p = 0.6 200 mg = −5.81 (−10.1, −1.5) p = 0.01 300 mg = −6.03 (−8.9, −3.2) p < 0.001	Not reported
2	NCT02160899 NCT02414594 (Viney, 2016)	64 participants to the phase 2 trial (35 in IONIS-APO(a)Rx and 29 in placebo in June 25, 2014, to Nov 18, 2015). 58 healthy volunteers to the phase 1/2a trial of IONIS-APO(a)-LRx (28 in sd group and 30 in md group in April 15, 2015, to Jan 11, 2016)	Adult	A: IONIS-APO(a)Rx 100 mg SC, once a week for 4 weeks, 200 mg SC, once a week for 4 weeks, then 300 mg SC, once a week for 4 weeks B: IONIS-APO(a)-LRx 6 doses of 10 mg, 20 mg, or 40 mg at days 1, 3, 5, 8, 15, and 22, for a total dose exposure in the active arms of 60 mg, 120 mg, or 240 mg per cohort	A: 51 B: 13	Placebo	A: 26 B: 3	A: day 85 or 99 B: day 30	A: reduction of Lp(a) plasma concentration B: reduction of Lp(a) plasma concentration	A: 66.8% (61.6, 72) B: 24·8% (3·1, 67·1) for 10 mg, 35·1% (2·2, 8·8) for 20 mg, 48·2% (10·9, 78·4) for 40 mg, 82·5% (50·5, 109·2) for 80 mg, 84·5% (65·2, 112·6) for 120 mg	There were two serious adverse events (myocardial infarctions) in the IONIS-APO(a)Rx phase 2 trial, one in the IONIS-APO(a)Rx and one in the placebo group, but neither were thought to be treatment related. 12% of injections with IONIS-APO(a)Rx were associated with injection-site reactions. IONIS-APO(a)-LRx was associated with no injection-site reactions.
3	European Clinical Trial Database 2012-004909-27 (Tsimikas et al., 2015)	healthy adults, BMI less than 32•0 kg/m(2), Lp(a) 25 nmol/L (100 mg/L) or more	18–65 years old	ISIS-APO(a)Rx, Single dose, SC injection 50 mg 100 mg 200 mg 400 mg ISIS-APO(a)Rx, Multi dose, SC injection 100 mg for a total dose exposure of 600 mg 200 mg for a total dose exposure of 1200 mg 300 mg for a total dose exposure of 1800 mg	3 3 3 3 8 8 8 7	Placebo	Single dose: 4 Multi dose: 6	day 30 day 36	Lp(A) reduction	Single doses of ISIS-APO(a)Rx (50–400 mg) did not decrease Lp(a) concentrations at day 30. Placebo outcome change from baseline: 5% (−8,15) Multidose ISIS-APO(a)Rx 100 mg 39.6%, p = 0.005 200 mg 59.0%, p = 0.001 300 mg 77.8%, p = 0.001	Mild injection site reactions were the most common adverse events. 2 volunteers excluded due to AE (one each in ISIS-APO(a)Rx 200 mg md (ec injection site adverse event) and 300 mg md (ec flu-like syndrome that resolved without sequelae). No SAE. Mild injection site reactions were the most common adverse events. ≥10% of participant in ISIS-APO(a)Rx group got headache and fatigue, no significantly different compared to Placebo.
4	Waldman, E (2017)	Established atherosclerosis, LDL ≥ 3.4 mmol/L (130 mg/dL) despite on stable maximal possible lipid lowering therapy for more than ≥ 3 months, BMI ≤ 40 kg/m2, women had to be postmenopausal or on highly effective contraceptive regimen, and fulfilled German criteria for lipoprotein apheresis	>18 yo, mean 42-72 yo	Mipomersen 200 mg, SC injection, weekly for 26 weeks (at least 12 weaks)	11	none	4	26 weeks, or between 12 and 26 weeks for discontinued patients (n = 4)	pre apheresis LDL cholesterol	−0.02 (−1.1, 1.1) p = 0.002	Of the 11 patients randomized to mipomersen, 3 discontinued the drug early due to side effects (2 for injection site reactions and 1 for ﬂu-like symptoms) and were replaced. Further 4 patients discontinued mipomersen during treatment weeks 12e26, again for side effects (1 due to elevations of liver enzymes, the other 3 due to moderate to rather severe injection site reactions (ISR) and ﬂu-like symptoms (FLS)) and were not replaced.
5	NCT01713361 (Büller et al., 2015)	undergoing elective primary unilateral total knee arthroplasty	18–80 years old	FXI-ASO, SC injection 200 mg 300 mg 9 times at day 1,3,5,8,15,22,29,36,39	134 71	enoxaparin 40 mg	69	3 months	Incidence of adjudicated total thromboembolism which was a composite of asymptomatic DVT, objectively confirmed symptomatic venous thromboemolism, fatal PE, unexplained death which PE could not be ruled out.	Efficacy (Total Venous Thromboembolism) 200 mg = −15 (−37, 7) p = 0.59 300 mg = −18 (−16, −29) p < 0.001	Total 12 AE (bleeding), 6 of which related to treatment.
6	Dasgupta et al. (2019)	Biopsy proven ATTR amyloidosis (hereditary or wild type) with clinical signs and symptoms of CHF (NYHA I - III), a left ventricular wall thickness ≥ 1.3 cm on TEE, stable renal function (GFR >35) and stable thyroid function (TSH <10 or normal serum T4)	No age restriction (mean 63.4–76.2 years old)	Inotersen 300 mg/1.5 ml subcutaneous/week	33	None	None	Every 6 months, MRI every year if there is no contraindication Still ongoing (3 years by the time this journal was published)	Decrease of LV mass (MRI), Decrease in left ventricular septal thickness (TEE), Increase of exercise tolerance (6MWT), Stable LVEF, Steady decline of BNP.	Total 8 AE, all related to treatment. AEs: Inflamation & Induration on the site of injection	
7	NCT01713361 (Tsimikas et al., 2020)	elevated screening plasma lipoprotein(a) level (≥60 mg per deciliter [150 nmol per liter]). Confounding factors: CAD, Overweigh, HT, DMT2, Familial Hypercholesterolemia, smoking	Adult 18–80 years old	APO(a)-LRx, SC injection 20 mg every 4 weeks, 40 mg every 4 weeks, 60 mg every 4 weeks, 20 mg every 2 weeks for 6 months	48, 48, 47, 48, 48	Placebo normal saline per week	47	6 months	percent change in Lipo(a) at 6 monts exposure, safety and efficacy	20 mg/4 weeks = 80.7 (1.2, 21) p = 0.003; 40 mg/4 weeks = 101.7 (7.3, 131.4) p < 0.001; 20 mg/2 weeks = 115.1 (9.8, 195) p < 0.001; 60 mg/4 weeks = 134.3 (24, 627.4) p < 0.001; 20 mg/1 weeks = 172.6 (109.3, 11.571)p < 0.001	Total 253 AE, 212 of which related to treatment. 2 deaths due to traffic accident and suicide. AEs:. influenza like symptoms, injection site reaction
8	Santos, Raul D (2015)	HoFH, Severe-HC, HeFH-CAD, HC-CHD	≥ 12 years old	Mipomersen 200 mg, SC injection, weekly for 26 weeks	Total 261 HoFH 51, Severe-HC 58, HeFH-CAD 124, HC-CHD 157	placebo	129	week 28 - week 28 + 24	LDL-C	HoFH = 0.3 (0.04, 0.6), p = 0.002; Severe-HC = 0.6 (0.4, 0.8) p = 0.002; HeFH-CAD = 0 (−0.2, 0.2) p = 0.001; HC-CHD = 0.6 (0.4, 0.8) p < 0.001	injection site reaction (+).
9	NCT00607373 NCT00706849 NCT00770146 NCT00794664. (Raal et al., 2010, Stein et al., 2012, Mcgowan et al., 2012)	HoFH, Severe-HC, HeFH-CAD, HC-CHD Comorbidites: smoker, metabolic syndrome, overweight-obese	n/a	Mipomersen 200 mg, SC injection, weekly for 26 weeks	382 HoFH 51, Severe-HC 57, HeFH-CAD123, HC-CHD 151,	placebo	126	week 28	Lp(a)	−26.4 (−32.1, −20.7) p < 0.001 median (interquartile range)	
10	NCT00770146 (thomas et al., 2013)	HC, CHD Comorbidities: DMT2	≥18 years old	Mipomersen 200 mg SC injection weekly, for 28 weeks	101	placebo	50	week 28 - week 24	LDL-C	−38 (−49.3824, −26.6176) p < 0.001	A total of 139 patietns experiencing AEs, 97 of which related to treatment. AEs: injectio site reaction, flu-like symptoms, ALD increased, hepatic stetosis
11	Luigetti, M et al. (2022)	hereditary aTTR	n/a	inotersen 14.6 ± 5.9 months (range, 6–24 months)	23	none	none	6–14.6 months	troponin, NTpro BNP, intervent septum thickness, BMI safety-- > number of dropouts	Troponin 0.01 (−0.0052, 0.0252) p = 0.19; NTpro BNP -45.6 (−703.82, 612.62) p = 0.88 IVS 1.5 (−0.46, 3.46) p = 0.12	5 dropouts, 2 of which related to treatment. 20 AEs are all related to treatment, which are: 4: severe thrombocytopenia 9: mid trombocytopenia 7: mild thrombocytopenia
12	Yang, X et al. (2016)	hyperTAG cohort 1: FCS cohort 2: hyperTAG of varying causes cohort 3: stable fibrate therapy	adult	Volanesorsen 100 mg, 200 mg, 300 mg weekly for 13 weeks	11, 13, 11	Placebo	16	176 days	apoCIII-apoB	apoCIII-ApoB 100 mg −31 (−17005, 16943) apoCIII-ApoB 200 mg 21026 (8505, 33547) p < 0.001 apoCIII-ApoB 300 mg −626803 (−640678, −612928) p < 0.001	not reported
13	Benson, MD et al. (2017)	hereditary and wild-type ATTR with moderate-severe cardiomyopathy biopsy-proven	adult/elderly >55 years old	IONIS-TTR])							10 patients experiencing AEs
14	Reeskamp, L et al. (2018)	high risk and severe HeFH persistent hyperchol maximal LDL-lowering therapy Comorbidities: Smoking Alcohol consumption HoFH CHD other atherosclerotic disease Hypertension DM statin	Adult>18 years old	Mimopersen 200 mg SQ 1x/week 70 mg SQ 3x/week for 60 weeks	104 102	Placebo	57 25	84 weeks	percent change LDL	−20.96 (−29.5085, −12.4115) p < 0.001 −18.80 (−20.7270, −16.8730) p < 0.001	A total of 259 AEs, 178 of which related to treatment
15	Sugihara, C et al. (2015)	dual chamber PPM and AF burden 1-10 comorbidities: Use of dabigatran/warfarin HT DM Hyperlipidemia Hypothyroidism Prior stroke Use of card meds as indicated above	adult >18 years old	ISIS-CRPRx 200 mg in 1 mL solution/SC in two injection 3x/wk for 1 week 1x/wk for 3 wks Total intervention: 4 weeks	7	none	none	every visit during drug administration; 4 week; and 8 week	change in AF burden before and after	MD: 1.6% (−1.45% to 4.65%) p = 0.37 CRP: -2.9 (−5.95, 0.15) mg/L p = 0.031	
16	NCT03728634 (Viney et al., 2020)	healthy, non-pregnant,/lactating, BMI <32, able to take vitamin A	18–65 years old	AKCEA-TTR-LRx (ION-682884)120 mg SD/SC 45 mg 4x dose/SC, 1x/month for 4 months 60 mg 4x dose/SC, 1x/month for 4 months 90 mg 4x dose/SC, 1x/month for 4 months	9 10 10 10	placebo	2 2 2 2		safety assesment: AEs-- > physical and lab findings PK parameters PD parameters	TTR SD: -80.40 (−94.0 to −66.8) 45 mg: -79.80 (−95 to −64.6), p < 0.001 60 mg: -84.60 (−98.9 to −70.3), p < 0.001 90 mg: -87.90 (−97.4 to −78.4), p < 0.001	A total of 7 AEs, 6 of which related to treatment.
17	NCT01737398 (Benson et al., 2018)	stage 1 and 2 hereditary TTR amyloidosis comorbidites: Val30Met TTR mutation stage 1 vs stage 2 Previous treatment with tafamidis and diflusinal	adults	inotersen 300 mg, SC injection, 3 injection for the 1st week, followed by weekly injection up to 65 wks (67 doses)	87	placebo	52	1 week after initiation 35 wks after initiation 66 week post treatment	“mNIS+7 score Norfolk QOLD-DN score"	mNIS+7: -19.70 (−21.3 to −18.1) p < 0.001 norfolk QOL-DN: -.17(xxxxx) p < 0.001	A total of 199 AEs, 119 of which related to treatment. 110→ any AEs 9→ serious AEs.

#### 3.1.2 Hypercholesterolemia

The most studied CVD risk factor is lipid disorders which cover a wide range of diseases (e.g. hypertriglyceridemia, hypercholesterolemia, elevated lipoprotein(a), etc.). Plasma triglycerides contain triglyceride-rich lipoproteins and lipoprotein particles. The elevation of plasma triglyceride-rich lipoproteins and the remnants is correlated to ASCVD. Its elevation also increases VLDL, chylomicron, and LDL-C, activating cholesteryl ester transfer protein. The liver produces very low-density lipoprotein particles. They contain apolipo-protein B-100, C-I, C-II, C-III, and E. LDL is a lipoprotein most abundant with apoB-100, a known modifiable risk factor of ASCVD targeted for therapies. Part of the LDL is lipoprotein (a), an LDL particle with apolipoprotein (a) attached to apo B-100 via a single disulfide bond [11,12]. ASOs target different checkpoints or proteins in lipid metabolism, such as apoCIII, apo(a), apo(b), and ANGPTL3 [13].

The liver produces the amino acid glycoprotein apoCIII, which is connected to apoB and HDL. ApoCIII affects serum triglyceride levels and obstructs the clearance of triglyceride-rich lipoproteins via both LPL-dependent and -independent pathways. Hence, when projected on LDL and HDL, the elevated level of apoC-III predicts skyrocketing CVD risk. Volanesorsen is a second-generation ASO directed to mRNA of apoCIII that has been proven to reduce plasma apoCIII and triglyceride levels by ∼70% in animal and human models. Phase II clinical research conducted by Yang et al. [14] later revealed that hypertriglyceridemic individuals had a significant reduction in apoC-III on apoB-100, apoCIII-Lp(a), and apo(a)-I lipoproteins (reduction of treatment arm vs placebo; 82.3 ± 11.7%, 81.3 ± 15.7%, and 80.8 ± 13.6% respectively, p < 0.001 for all). This study implies the potency of Volanesorsen to reduce triglycerides and apoC-III-mediated cardiovascular risk.

Another important particle in lipid metabolism is apoB, an essential structural protein of all atherogenic lipoproteins (i.e. LDL-C, IDL-C, VLDL-C, Lp(a)). Mipomersen, an ASO that inhibits the synthesis and secretion of apoB, has been widely studied for the past decade [15,16]. Mipomersen binds to the apoB mRNA in the liver, causing mRNA degradation and, as a result, the cessation of apoB protein translation [17]. Several phase III trials have been conducted in different populations. Thomas et al. [8] tested mipomersen in a population of non-familial hypercholesterolemia with high CVD risk due to coronary heart disease or type 2 diabetes mellitus (T2DM), which resulted in a significant reduction of LDL-C (mean difference treatment vs placebo, −38 95% CI -49.3824 to −26.6176, p < 0.001). The apoB and Lp(a) also decreased in number. However, 96% (97/101) of subjects experienced mild adverse events related to treatment (e.g. injection site reaction, flu-like symptoms to hepatic steatosis) [13]. Santos et al. [18] conducted four mipomersen phase III trials in people with severe hypercholesterolemia, homozygous familial hypercholesterolemia (FH), heterozygous FH with concurrent coronary artery disease (CAD), and hypercholesterolemia with a high risk for CAD. All his studies reported that mipomersen significantly reduced all LDL particles and Lp(a) concentrations, with an adverse event of injection site reactions. Previously, three phase II studies reported a similar trend of decreased concentration of apoC-III, LDL-C, and apoB [15,16,19]. However, the rate of side effects was high, ranging from mild to moderate adverse events (i.e. site injection reactions, flu-like symptoms, and elevated liver enzymes).

#### 3.1.3 Atrial fibrillation

Atrial fibrillation is a common cardiac arrhythmia that involves complex pathophysiology, of which the exact etiology remains unclear. Inflammation is believed to play a significant role in the development of AF. Thus, the reduction of any inflammatory markers has been found to suppress AF development [7]]. A second-generation 2′-O-(2-methoxyethyl) chimeric antisense oligonucleotide (ASO) called ISIS-CRPRx (ISIS-329993) inhibits CRP in order to function. CRP is an acute-phase protein that was proposed to be able to promote atrial fibrosis, oxidative stress, and electrical remodeling. Tanaka et al.'s cohort study found a strong correlation between raised hs-CRP levels and an increased risk of atrial fibrillation in the Japanese population [20]. Similar cohort research conducted by Lee Y et al.‘s discovered that chronically increased CRP levels independently predicted the development of AF [21]. In a phase II RCT by Sugihara et al. [22] this medication has been utilized for AF patients with dual chamber permanent pacemakers (PPM) and was generally well tolerated with no significant side events in the study population. However, the study yielded no significant result in reducing AF burden (treatment arm vs. placebo: OR 1.6, 95% CI −2.42 to 5.62, p = 0.37). Nonetheless, this drug significantly reduced CRP levels by 63.7% (*relative reduction*; 95% CI 38.4–78.6%, p = 0.003). A previous phase I RCT by Noveck et al. [23] also revealed that pre-treatment with ISIS-CRPRx in endotoxin-induced healthy subjects attenuated CRP levels by 37% (400 mg dose) and 69% (600 mg dose; p < 0,05 vs. placebo). There is no direct causal pathogenic role of CRP in AF, consequently this contributed to imperceptible clinical outcomes. Secondly, on average, the study population of phase II RCT had low baseline levels of CRP. The impact of lowering higher levels of CRP on AF warrants further investigation.

#### 3.1.4 The use of ASO in venous thromboembolism

Factor XI Antisense Oligonucleotide (FXI-ASO) demonstrated a positive outcome in one phase II RCT by Büller et al. [24] in the prevention of venous thrombosis. FXI-ASO (ISIS 416858) is a second-generation ASO, which works by inhibiting Factor XI production, thereby hampering the blood coagulation process. A total of 281 subjects who underwent total knee arthroplasty were randomized to receive either 200 mg FXI-ASO, 300 mg FXI-ASO, or 40 mg Enoxaparin as a comparison. Subjects in the treatment arms received either 200 or 300 mg of FXI-ASO, while subjects in the comparison arm were given 40 mg of Enoxaparin. The result showed that 300 mg of FXI-ASO had a significantly lower incidence of venous thromboembolism compared to the Enoxaparin group (FXI-ASO vs. Enoxaparin; n = 3 [4%], 95% CI 1 to 12 vs. n = 21 [30%], 95% CI 20 to 43, p = <0.001) with a risk difference of −26 (upper limit of 95% CI -16). On the other hand, 200 mg of FXI-ASO did not show any superiority compared to Enoxaparin (p = 0.59). Serious adverse events occurred in four subjects and two subjects needed to discontinue the use permanently due to worsening arterial hypertension and bleeding from the surgical site [24].

#### 3.1.5 The use of ASO in ATTR

Amyloidosis is a disease caused by a buildup of an abnormal protein named amyloid in body organs [25]. Transthyretin protein, or TTR, misfolds, reshapes, and forms fibrous clumps that are deposited in many parts of the body, resulting in ATTR amyloidosis, a serious systemic illness [26]. Most patients manifest signs and symptoms of nerves and heart, although other organs may be involved. ATTR can be inherited in an autosomal dominant fashion. According to research by Tanskanen et al., 25% of myocardial samples from individuals older than 85 years old showed ATTR amyloid deposits [27]. Restrictive cardiomyopathy is caused by myocardial ATTR amyloid deposition and is characterized by the thickness of the biventricular wall, stiffness of the myocardium, and the emergence of systolic and diastolic dysfunction. Recently, pharmacological therapy to improve outcomes and prolong survival has emerged. Reducing TTR synthesis and interrupting the appropriate mRNA by preventing dissociation into amyloidogenic monomers or cleavage into amyloidogenic fragments are two therapeutic methods that are intended to lessen continuing ATTR amyloid fibrillogenesis [28,29]. One of which is ASOs targeting mRNA of mutant transthyretin genes to suppress the hepatic production of TTR called Inotersen or IONIS-TTRRx [25]. An identical sequence and similar design of ASO targeting TTR mRNA is AKCEA-TTR-LRx, whose particles are conjugated to a GalNAC3 ligand to accelerate hepatocyte uptake [30]. When ASO binds to complementary mRNA, it causes the mRNA to degrade in several different ways, including sterically inhibiting ribosome attachment and encouraging endogenous ribonuclease H1-mediated breakdown. Without a transfection reagent, ASO can penetrate cells [28]. Phase III trials of inotersen in ATTR patients with polyneuropathy reported an improved quality of life with several adverse events ranging from glomerulonephritis to thrombocytopenia and death. Out of 5 deaths, one was related to grade 4 thrombocytopenia, while the others were unrelated to therapy [30]. Phase II trials of inotersen yielded a similar trend in which disease progression is limited and cardiac amyloid burden reduced. This trial did not record adverse renal effects; however, one sudden cardiac death post-emergency cholecystectomy was reported [31]. The phase I trial reported several adverse events, such as decreased renal function (one patient) and declined platelet level, although no grade 4 thrombocytopenia. The cardiac parameters measured by left ventricular function and cardiac MRI were found to be constant or increased [32]. The effect of lowering TTR on cardiac amyloidosis warrants further investigations. No serious adverse events were recorded during the AKCEA-TTR-LRx phase I trial, demonstrating the drug's safety; additionally, there was a significant drop in TTR levels. Further study is required to evaluate the efficacy of AKCEA-TTR-LRx [33].

### 3.2 Aptamer

**Fig. 3:**
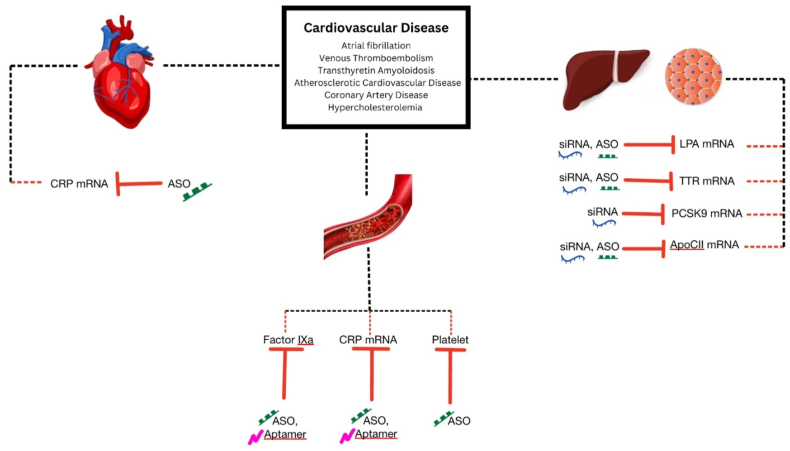
ASO, Aptamer, and siRNA in the currently accepted molecular disease model of CVDs and risk factors.

Globally, cardiovascular disease has a high mortality rate, which requires the development of novel diagnostic and therapeutic methods. Aptamers are single-stranded oligonucleotides that can compete with antibodies in cardiac applications as they uniquely recognize and bind targets by forming unique structures *in vivo* [34]. Major cardiovascular events are frequently caused by thrombus development. Therefore, the alternative treatment option might involve using RNA aptamers as a reversible FIXa inhibitor [6]. A novel RNA-aptamer-based FIXa inhibitor is named pegnivacogin (RB006) [35]. An ASO, anivamersen (RB007), improves hemostasis by blocking pegnivacogin from binding to Factor IXa [35] (Fig. 3).

Antiplatelet therapy is used to treat ischemic disorders, with the main emphasis being on the activation and aggregation of platelets rather than the activity of von Willebrand Factor (vWF) [6,36]. However, since vWF is an important factor in atherogenesis and artery circulation, it may be a target for cardiovascular disease intervention. vWF has a unique role in thrombus generation, and as an aptamer targeting molecule, has shown early success in antithrombotic treatment [6]. The GPIb-vWF pathway has been blocked by anti-vWF aptamers [37]. Without causing significant bleeding, ischemia events, or other severe negative effects, factor IXa was successfully inhibited with an RNA aptamer, and its coagulant activity was actively restored with an antidote [35].

#### 3.2.1 The use of aptamer in healthy volunteers

Staudacher et al.‘s study [35] found that the new RNA-aptamer-based FIXa inhibitor pegnivacogin reduced platelet reactivity in blood samples from healthy volunteers. In this work, platelet aggregation calculations were used after ex-vivo incubation with pegnivacogin [35]. According to Arzamendi et al. [38], platelet adhesion is reduced by ARC1779, an aptamer to the VWF A1 domain, in healthy subjects without having a meaningful impact on P-selectin or vWF expression. However, there were no differences in aggregation between ARC1779 and the placebo group in healthy volunteers. After perfusion, platelet activation was measured using blood from healthy volunteers in their research [38].

#### 3.2.2 The use of aptamer in CAD patients

Our systematic literature search has indicated five studies on the use of aptamer to treat CAD. One study was conducted using an ex-vivo model (human blood), and the others used an in-vivo model (human). Four studies used pegnivacogin and anivamersen reversal [35,[39], [40], [41]]. According to a study by Posvic et al. [39], using these medications with at least 50% reversal demonstrated safety compared to the Heparin group. This RNA aptamer, pegnivacogin, is a secure anticoagulation method for invasively managed ACS patients. It was reported that its safety was likely due to the lower incidence of major bleeding and ischemic events because pegnivacogin is known as a selective factor IXa inhibitor [39]. The effect of pegnivacogin on activated platelets could be negated by ASO, anivamersen (RB007). In their study, Cohen et al. [40] also noted that an active reversal technique using RB007, followed by an anticoagulation strategy utilizing RB006 to suppress factor IXa, exhibits a balance of safety and efficacy following PCI.

Studies by Staudacher et al. and Chan et al. [35,41] used Pegnivacogin (RB006) as the active drug and RB007 as the antidote. Both approaches reduced the aggregation of thrombocytes in the pegnivacogin group compared to the placebo group. Pegnivacogin has been shown to decrease platelet aggregation in the blood of healthy individuals and in patients with acute coronary syndrome by inhibiting the formation of thrombin and lowering platelet reactivity, by indirect thrombin reduction. Pegnivacogin inhibits factor IXa by decreasing platelet activation and aggregation in vitro (Table 2) [35]. Chan et al. [41] showed that RB006 increased activated partial thromboplastin time in a dose-dependent manner, whereas RB007 reversed activated partial thromboplastin time to baseline values without serious side effects. The most common side effect was mucocutaneous bleeding, which occurred in 10% of participants.

**Table 2:** Aptamer.

No	Study registration number (ref)	Study Population	Age Group	Intervention Arm	n	Comparator arm	n	Time to follow Up	Primary Endpoint	Outcome: Mean Difference (Treatment vs Placebo)	Adverse Events
1	10.1093/eurheartj/ehs232 NCT00932100 (Povsic, TJ et al., 2013)	Non-ST-elevation ACS patients with planned early cardiac catheterization via femoral access <24h. Past medical history of CHF, MI, Previous PCI, Previous CABG, HTN, T2DM, Renal Insuficiency, Stroke, Current tobacco use.	25–75 years old	Pegnivacogin 1 mg/kg and Anivamersen reversal 0.075, 0.20, 0.40, 1.00 mg/kg	REG1 25–100% (n = 40,113,119,194)	Heparin	161	30 days	Primary endpoint: total ACUITY bleeding. Secondary endpoints: major bleeding and ischaemic event.	Total bleeding (%) 33.7, 2.7, 3.7, −1.3; Major bleeding (%) 10, 1, −2, −3; Ischaemic event (%) −2.7 (0.2,1.4)	REG1 n = 60. Heparin n = 55. 3 incidence of allergic-like adverse events within 24 h of drug administration, 2 of 3 are SAE. REG 1 (hives 0.2%, hypotension 2.4%, rash 0%, dyspnoea 0.9%). Heparin (hives 0%, hypotension 1.9%, rash 0.7%, dyspnoea 0%)
2	10.1177/1076029610384114 (Arzamendi D et al., 2011)	CAD patient on double antiplatelet therapy and normal volunteers (CAD patient n = 27 (Male (n = 22), Hypertension (n = 9), Hypercholestrolemia (n = 16), T2DM (n = 4), Smoker (n = 10), ACS (STEMI n = 17; NSTEMI n = 1; UA = 9)), Healthy volunteers n = 5)	18–75 years old	exvivo treated pretherapy (incubated 5 min before the onset of perfusion) or 10 min posttherapy on damaege arteries with: ARC1779 (25, 83, and 250 nmol/L), or Abciximab (100 nmol/L), or placebo	n = 27	placebo	n	15 min	Platelet function	Platelet adhesion: Pretherapy with ARC1779 or Abciximab in patients taking ASA and CPG (n CAD = 17) effect on platelet adhesion (unit: platelets × 10^6/cm2). ARC1779 83 nmol/L: 4.8 (p < 0.05) ARC1779 250 nmol/L: 3.8 (p < 0.05) Abciximab 100 nmol/L: 2.9 (p < 0.05) Placebo: 7.3 (p < 0.05) Pretherapy with placebo in healthy patient (n = 5) effect on platelet adhesion: 81.9 ± 23.6 × 10^6 platelets/cm2 Posttherapy with ARC1779 in CAD (n = 10) p > 0.05 in platelet adhesion compared to placebo. Platelet aggregation Abciximab abolished platelet aggregation in response to TRAP-1, ADP, and AA both in healthy volunteers and in patients. Collagen-induced aggregation: in healthy volunteers; averaged 18 Ω reduce to 2 Ω with abciximab, but unaffected by ARC1779 (18 Ω) in CAD patient; reduced to 3 Ω by abciximab, and unaffected by ARC1779 (4 Ω) Platelet activation This was done on blood samples from healthy volunteers after the perfusion experiments. Neither abciximab nor ARC1779 has a significant effect on P-selectin or vWF expression. Platelet−leukocyte binding increased after blood perfusion (control) compared with nonperfused blood (baseline), not significantly affected by ARC1779 or abciximab.	
3	10.1177/2048872617703065 (Staudacher DL et al., 2019)	Healthy volunteers and patient with ACS	≥18 years old (whole blood sample)	Pegnivacogin or Pegnivacogin 1 mg/kg + Anivamersen (RNA Aptamer reversal agent)	n	Placebo	n	20 min	CD62P-expression, PAC-1 binding	Pegnivacogin when compared with placebo CD62P expression 20 mikroM ADP (n = 9): -13.38 p = 0.027 1 mikroM ADP (n = 24): -6.59 p = 0.031 PACbinding 20 mikroM ADP (n = 11): -16.98 p = 0.0098 1 mikroM ADP (n = 25): -9.59 p = 0.0008 Pegnivacogin effect on ADP-activated platelets could be completely negated by Anivamersen, compared with Placebo CD62P expression 20 mikroM ADP (n = 10): -2.42 p = 0.922 1 mikroM ADP (n = 3): -2.38 p = 0.449 Blood from healthy subject after ex-vivo incubation with 150 μl pegnivacogin: platelet aggregation −3.66% p = 0.002, n = 10 Patient CAD treated dual antiplatelet after 20min iv 1 mg/kg pegnivacogin: platelet aggregation −56.79% p = 0.020, n = 3	
4	10.1161/CIRCULATIONAHA.107.745687 (Chan, MY et al., 2008)	Subjects with stable CAD	50–75 years old	aptamer (RB006) sd 1 min iv ASO (RB007) sd 3h iv	Group 1 = 28; 6,6,8,8 (RB006 15,30,50,75 mg with RB007 30,60,100,150 mg) Group 2 = RB006+Placebo antidote = 14; 3,3,4,4	placebo	8	day 7	safety, tolerability, pharmacodynamic	RB006 increased the activated partial thromboplastin time dose dependently; the median activated partial thromboplastin time at 10 min after a single intravenous bolus of 15, 30, 50, and 75 mg RB006 was 29.2, 34.6, 46.9, and 52.2 s, P < 0.0001. RB007 reversed the activated partial thromboplastin time to baseline levels within a median of 1 minute with no rebound increase through 7 days.	No major bleeding. 10% experienced non–dose-dependent and mainly mucocutaneous bleeding.
5	NCT00715455	undergo non-urgent PCI have a prior indication for PCI pre-treatment with aspirin and CPG	18–80 years old	RB006 1 mg/kg/IV, SDRB007 0.2:1 (50% efficacy)/2:1 (100%), SD	20	UFH IV treated	4	48 hours 14 days	major bleeding 48 h/hospital discharge all-cause death, MI-event, urgent revasc 14 days	1.0 Median (0.9, 1,1) p < 0.001	A total of 4 AEs, 2 patients from treatment group2 patients from control/comparison group

The fourth study in the field of CAD indicated by our literature search involved the use of ARC1779 to examine its efficacy. ARC1779 has a rapid antiplatelet action. ARC1779 reaches maximum concentration level within 7–30 min [38]. The study by Arzamendi et al. demonstrated that giving ARC1779 as a first medication had an additive impact on the dual antiplatelet therapy by considerably lowering platelet adhesion in comparison to placebo [38]. Even in patients who had previously had aspirin and clopidogrel treatment, ARC1779 significantly decreased platelet adhesion but not platelet aggregation [38]**.**

### 3.3 siRNA

#### 3.3.1 Definition and mechanism of action

Small interfering RNA (siRNA), silencing RNA or short interfering RNA, is a class of double-stranded (ds) RNA molecules with a length of about 20–25 nucleotides, which have various biological functions. One of its main roles is the RNA interference (RNAi) pathway, which destroys selective mRNAs by silencing or downregulating the expression of target genes [42].

RNAi's first step involves the cleavage of longer dsRNA molecules to shorter siRNAs, which typically have a dinucleotide overhang at the 3′ end of each strand. This process is mediated by an RNase III-like enzyme called Dicer. Once siRNAs are formed, they bind to a multiprotein complex called RISC (RNA-induced silencing complex). The siRNA strands then dissociate in the RISC complex and the more stable strand at the 5′ end usually integrates into the active RISC complex. The single-stranded antisense siRNA component is responsible for directing and targeting the RISC complex to the target mRNA. Finally, the mRNA is cleaved with the help of catalytic RISC proteins of the Argonaute family (Ago2) [43]. Table 3 illustrates the use of siRNA as therapy in studies of CVDs and risk factors, drug monitoring, and outcomes, including death, MACE, and allergic reactions.

**Table 3:** siRNA.

No	Study registration number (ref)	Study Population	Age Group	Intervention Arm	n	Comparator arm	n	Time to follow Up	Primary Endpoint	Outcome: Mean Difference (Treatment vs Placebo)	Adverse Events
INCLISIRAN											
1	NCT01437059 (Fitzgerald et al., 2014)	Healthy adults with LDL-C higher than 3.00 mmol/L	18–65 years old	ALN-PCS one dose IV 0.015 mg/kg 0.045 mg/kg 0.090 mg/kg 0.150 mg/kg 0.250 mg/kg 0.400 mg/kg	Total 24 3 3 3 3 6 6	Placebo (NS)	8	Data for adverse event: 28 days Other data: 180 days	Safety, tolerability, and adverse event	Mean percentage change vs placebo: PCSK9 (−45.3, −86.0, −71.5, −96.2, −98.3, −114.5); LDL-C (−6.6, −13.4, −27.2, −24.0, −30.1, −47,2)	Treatment-emergent adverse events (TEAE) (rash, headache, hiccups, cold symptoms, paraesthesia, polyuria or disuria, infusion-site hematoma) ALN-PCS n = 19 (79%) Placebo n = 7 (88%)
2	NCT02314442 (Fitzgerald et al., 2016)	Healthy volunteers with LDL cholesterol level ≥ 100 mg/dl, TG level ≤ 400 mg/dl	18–65 years old sd phase, 18–75 years old md phase	Single dose phase: sc inclisiran (n = 4 each) 25 mg 100 mg 300 mg 500 mg 800 mg (two cohorts for the 800-mg dose). Multi-dose phase: (n = 4-8 each) 125mg/w for 4 weeks, 250mg/2 weeks for 4 weeks, 300 mg/month for 2 months with and without statin, 500 mg/month for 2 months with and without statin	SD 4 each MD 4-8 each	Placebo	SD n = 6, MD phase n = 11 (8 in md phase without statin group and 3 in md phase with statin group)	56 days for sd phase, ≤84 days for md phase. PD end point were evaluated for an additional month (until 180 days after last dose of therapy) after completion of safety and side effect profile assessment	Safety, side effect profile	sd phase: PCSK9 (−46.0; −31.4; −73.9; −69.3; −72.5) sd phase: PCSK9 (<0.05; n/a; <0.001; <0.001; 0.001)	No SAE, most common adverse events were cough, musculoskeletal pain, nasopharyngitis, headache, back pain, and diarrhea. in sd phase (≥5% participant in inclisiran group): 2 of 18 cough, musculoskeletal pain, nasopharyngitis. In md phase (≥10% participant in inclisiran group) 6 of 33 headache, 5 (15%) diarrhea, 5 (15%) back pain, 4 (12%) nasopharyngitis
3	NCT02597127 (Ray et al., 2017)	LDL >70 mg/dL for patient with history ASCVD/>100 mg/dL without history ASCVD max statin therapy	62–74 years old	SD: - Placebo - 200 mg - 300 mg - 500 mg DD: - Placebo - 100 mg - 200 mg - 300 mg	370	Placebo	127	Primary 180 days Other data 210–240 days	Percentage change from baseline in LDL-Cholesterol level	Serious Adverse events Intervention group SD 200 mg 6 (10%); 300 mg 5 (8%); 500 mg 6 (9%); DD 100 mg 11 (18%); 6 (10%); 7 (11%) Placebo SD placebo 3 (5%); DD placebo 6 (10%)	
4	NCT03397121 (Raal et al., 2020)	Diagnosed with heterozygous familial hypercholesterolemia LDL at least 100 mg/dL despite max statin therapy	47–64 years old	Inclisiran SC 300 mg Day 1, 90, 270, 450	242	Placebo	240	Day 30, 150, 330, 510, 540	I = Percentage change from baseline LDL - C levels on day 510 II = time-averaged percent change in the LDL - C level between day 90 and day 540	I = −47.9% (95% CI, −53.5 to −42.3; P < 0.001) II = −44.3% (95% CI, −48.5 to −40.1; P < 0.001)	Patients with ≥ 1 serious adverse event - Intervention group 18 (7.5%), 1 death from cardiovascular cause 1 (0.4%) - Control 33 (13.8%)
5	NCT03400800 (Ray et al., 2020)	high risk, primary prevention patients or those with ASCVD (secondary prevention)	≥18 years old	Inclisiran 300 mg SD SC Day 1, 90, 270, 450	98	Placebo	105	up to day 540	Percentage change in LDL-C from baseline at day 510 and time adjusted percentage change in LDL-C from baseline after day 90 and up to day 540	LDL-C changes from baseline to day 510 (−43.7%). Time adjusted change in LDL-C from baseline after day 90 up to day 540 (−41.0%). Absolute change difference of LDL-C is -.5 mmol/dL (−58.4 mg/dL) between groups P < 0.0001, <0.0001, <0.0001	SAE, AE at injection site Intervention: SAE (n = 20), AE at injection site (n = 4) Control: SAE (n = 13), AE at injection site (n = 0)
6	Ray et al. (2022)	ITT Population (For efficacy analyses: Pooled analysis of ORION-9,-10 and −11, included patients with heterozygous familial hypercholesterolaemia, atherosclerotic CV disease (ASCVD), or ASCVD risk equivalent on maximally tolerated statin-therapy Population (for Safety analyses) : All patients who received at least 1 dose of Inclisiran/placebo	54–73 years old	Inclisiran 284 mg SD SC Day 1, 90 and 6-monthly until 18months	1833	Placebo	1827	540	Change in LDL- C Levels & Safety population (risk of cardiovascular events)	Change in LDL-C levels day 90: - 50.6% [95% CI (−52.3 to −49.0); P < 0.0001] day 540: - 51.4% [95% CI (−53.4 to −49.4); P < 0.0001] Inclisiran significantly reduced MACE (OR[95%CI]: 0.74 [0.58–0.94]), but not fatal and non-fatal MI (OR [95% CI]: 0.80 [0.50−1.27]) and fatal and non-fatal stroke (OR [95% CI]: 0.86 [0.41−1.81]).	- MACE Intervention: 131 Control:171 - Fatal and non-fatal MI Intervention: 33 Control:41 - Fatal and non-fatal stroke Intervention: 13 Control:15
7	NCT03399370 and NCT03400800 (Ray et al., 2020)	ASCVD, LDL >70, statin and lipid lowering therapy use, GFR >30	adult >18 years old	Inclisiran 284 mg/SC	ORION 10 : 781 ORION 11 : 810	Placebo	780 807	day 30 day 150 day 330 day 510 day 540	Percentage change in LDL time adjusted LDL change (throughout the follow-up period)	−52.3 P < 0.001 −49.9 P < 0.001	Total AEs: 1156 Serious AEs: ORION 10: 175 ORION 11: 181
8	NCT02597127 and NCT03159416 (Wright et al., 2020)	Participant with normal renal function and mild, moderate and severe RI from phase 1 ORION -7 renal study and the phase 2 ORION-1 study; BMI 18–40kg/m2 and BW > 50 kg	18 < age <80 years old	(ORION 7) = SD Placebo and 300 mg; DD Placebo and 300 mg (ORION 1)1 = Normal Function, 2 = Mild RI, 3 = Moderate RI, 4 = Severe RI	ORION 1 = 122 ORION 7 = 31	Placebo	ORION 1 = 125 ORION 7 = 0	180 days atau max 360 days	ORION 7: PK, Safety, PD ORION 1: PK, Safety	Participants with at least 1 TEAE (treatment-emergent adverse event) Intervention: 126 Control: 101	
9	Raal et al. PMID: 36217872	HeFH ASCVD ASCVD-risk equivalent	adults	Inclisiran 284 mg/SC	148	Placebo	150	510 days	LDL percentage change at day 510 time-averaged percentage LDL change	−54.2% P < 0.0001	Participants with ≥1 TEAEs: 233 Serious TEAEs: Intervention: 32 Placebo: 37
PATISIRAN											
1	NCT01148953 and NCT01559077 (Coelho et al., 2013)	ALLN 01:biopsy-confirmed TTR amyloidosis mild-moderate neuropathy karnofsky performance status >60 BMI 18.5–33 NYHA II or less adequate liver, renal, thyroid function not pregnant/childbearing potential ALLN 2: 18-45, healthy, BMI 18–31.5.	adults >18 years old	ALN-TTR01 (0.01–1.0 mg/kg) - IV ALN-TTR02 (0.01–0.5 mg/kg) - IV	24 13	Placebo	8 4	70 days	reduction in TTR level	−38% (dosage 1.0 mg/kg) P 0.01 ALLN 02 0.15 = −85.7% P 0.001 0.3 = −87.6% P 0.001 0.5:= −93.8% P 0.001	ALN-TTR01 Infusion Reaction: 5 Fatigue: 2 Placebo: 0 ALN-TTR02 Skin Erythema: 6 Infusion Reaction: 1 Placebo: 2 (Skin Erythema)
2	NCT01961921 (Coelho 2020)	hATTR amyloidosis	29–77 years old	Patisiran 0.3 mg/kgbb - IV once/3 weeks	Patisiran alone (n = 7)	Patisiran + TTR tetramer stabilizer	n = 19	24 months	The primary objective was to evaluate the safety and tolerability of long-term dosing with patisiran.	(-0.28)	SAE 7, death 2, any AE leading to discontinuation 2. None of which were considered related to Patisiran flushing (n = 7), infusion-related reactions (n = 6), diarrhea (n = 3)
3	NCT01960348 (Solomon et al., 2019)	Patients with hereditary transthyretin-mediated (hATTR) amyloidosis with polyneuropathy and cardiac amyloidosis	61 (54-67) years old	Patisiran 0.3 mg/kgbb - IV once/3 weeks	90 (71.4%) patisiran	Placebo	36 (28.6%) placebo	18 months	improved left ventricular (LV) global longitudinal strain (LV GLS)	Patisiran improved the absolute GLS (least-squares mean [SE] difference, 1.4% [0.6%]; 95% CI, 0.3%-2.5%; P = 0.02) compared with placebo at 18 months, with the greatest differential increase observed in the basal region (overall least-squares mean [SE] difference, 2.1% [0.8%]; 95% CI, 0.6%-3.6%; P = 0.006) and no significant differences in the mid and apical regions among groups	
4	NCT01960348	Had a diagnosis of hATTR amyloidosis with a documented TTR mutatiom and symptomatic neuropathy, were ambulatory, had adequate liver function and adequate renal function, and were included in the prespecified cardiac subpopulation (baseline LV wall thickness >13 mm, no history of aortic valve disease or hypertension) Enrolled participants which did not fulfill criteria to be included in prespecified cardiac subpopulation	18–85 years old	Patisiran 0.3 mg/kgbb - IV once/3 weeks	Cardiac subpopulation 90	Placebo	36	Echo parameters at 18 months NTproBNP and 10MWT gait speed at 9 months and 18 months	Reduction of left ventricular wall thickness, interventricular septal wall thickness, posterior wall thickness, and relative wall thickness. Increase of end diastolic volume, decrease of global longitudinal strain, increase of cardiac output. Decrease of NT pro BNP. Increase of 10MWT gait speed. No significant outcome in echo parameters. Decrease of NTproBNP. Increase of 10MWT gait speed.	Reduction in mean LV wall thickness (least-squares mean difference±SEM, −0.9 ± 0.4 mm; P = 0.017) was observed with patisiran compared with placebo. In patisirantreated patients compared with placebo, global longitudinal strain was decreased (−1.4% ± 0.6%, P = 0.015), cardiac output was increased (0.38 ± 0.19 L/min, P = 0.044), and LVEDV was increased (8.31 ± 3.91 mL, P = 0.036) Patisiran reduced NT-proBNP compared with placebo at 9 months (ratio of fold change patisiran/placebo, 0.63; 95% CI, 0.50–0.80) and 18 months (ratio of fold change patisiran/placebo, 0.45; 95% CI, 0.34– 0.59; P = 7.7 × 10–8), corresponding to a 55% reduction relative to placebo	Cardiac serious adverse events Intervention: 20 Control: 10
5	NCT01960348	hereditary transthyretin amyloidosis with polyneuropathy, some patients has cardiac abn (NYHA I dan II)	18–85 years old	Patisiran 0.3 mg/kgbb - IV once/3 weeks	148	Placebo	77	18 months	mNIS+7, LVthickness, LVLStrain	−34; −0.9; −1.37 <0.001; 0.02; 0.02	diarreha, edema, nausea, cough, asthenia, death, etc Intervention: 143 Control: 75
6	NCT01960348	Hereditary transthyretin amyloidosis with polyneuropathy, some patients has cardiac abn (NYHA I dan II)	n/a	Patisiran 0.3 mg/kgbb - IV once/3 weeks	148 (90 yg cardiac)	Placebo	77 (36 yang cardiac)	18 months	measures of overall QoL	AEs not reported	
REVUSIRAN											
1	NCT02319005 (Judge et al., 2020)	TTR mutation and amyloid deposits, hx of hf and cardiac involvement on echo	18–90 years old	Revusiran 500mg/SC	140	Placebo	66	18 months	6MWT, Troponin I, NTpBNP	the study was prematurely discontinued due to an imbalance of deaths observed in the revusiran group (18 patients, 12.9%) compared with the placebo group (2 patients, 3.0%) during the on-treatment period	
SLN360											
1	NCT04606602 or 2020-002471-35 (Nissen et al., 2022)	no known CVDs Lp(a) conc >150 nmol/L BMI 18–45 kg/m2	adults 36–63 years old	SLN360 SD 30 mg/SC 100 mg/SC 300 mg/SC 600 mg/SC	24 (6 each dose)	Placebo	8	150 days	Safety and tolerability	Participants with any treatment-emergent adverse event intervention group: 100% placebo group: 75%	

#### 3.3.2 The use of siRNA in healthy volunteers with elevated Lipoprotein(a)

Lipoprotein(a) has been previously known as an independent risk factor for atherothrombotic cardiovascular disease, which is genetically determined. Higher levels of Lp(a) have been linked in studies to an increased risk of stroke, myocardial infarction, and peripheral arterial disease (PAD) [44,45].

While some lipid-modifying treatments, such as niacin or PCSK9 inhibitors, have demonstrated moderate ability to reduce Lp(a) levels, no medication has yet been approved to treat elevated Lp(a) concentrations. The current study investigated different approaches to reduce Lp(a) levels, including the use of oligonucleotide therapies, among which SLN360 was included [44].

SLN360 is a specific kind of double-stranded, 19-nucleotide siRNA that has been covalently joined to a tri-antennary N-acetyl-galactosamine (GalNAc) moiety and chemically stabilized. The LPA gene, which codes for apolipoprotein(a) (apo[a]), a vital and rate-limiting component in the hepatic manufacture of the Lp(a) particle, has been specifically targeted by the treatment [44,45].

A study by Nissen et al. [44] investigated the effects of siRNA SLN360 on individuals who had elevated lipoprotein(a) levels but no prior history of cardiovascular disease. The results showed that SLN360 was generally well-tolerated, with only mild treatment-emergent adverse events reported, such as injection site reactions and headaches. None of these adverse events caused participant withdrawal. Of the 32 participants, only one experienced two serious adverse events, including hospitalization for post-SARS-CoV-2 vaccination headache and cholecystitis complications, which were considered unrelated to the study drug [44].

This study also demonstrated a dose-dependent decrease in plasma Lp(a) levels. The maximum median change in Lp(a) levels from baseline to day 150 was −20 (IQR, −61 to 3) nmol/L, −89 (IQR, −119 to −61) nmol/L, −185 (IQR, −226 to −163) nmol/L, −268 (IQR, −292 to −189) nmol/L and −227 (IQR, −270 to −174) nmol/L placebo and SLN360 30 mg, 100 mg, 300 mg and 600 mg [44].

#### 3.3.3 The use of siRNA in hypercholesterolemia and heterozygous familial hypercholesterolemia

High levels of low-density lipoprotein (LDL) cholesterol are important risk factors for the development of atherosclerotic cardiovascular disease. The risk of coronary heart disease changes by around 30% for every 30 mg/dL (0.78 mmol/L) variation in LDL cholesterol. Although statins have been proven to be effective in reducing LDL cholesterol levels, there is substantial variation in individual responses to these drugs, leading to the need for newer therapies [[46], [47], [48]].

Proprotein convertase subtilisin type 9 (PCSK9) is a newly identified target for lowering LDL cholesterol levels. Mutations leading to loss of function in PCSK9 are associated with lower circulating LDL cholesterol levels and reduced risk of cardiovascular disease [46,47]. Inclisiran (previously known as ALN-PCS) is an investigational, long-acting, chemically synthesized siRNA molecule directed against PCSK9 [47].

Inclisiran was shown to be safe in a phase I clinical trial in healthy volunteers, with all reported side effects being mild or moderate. No serious adverse events were drug-related or resulted in participant withdrawal from the study [46,47]. A phase II study evaluated the efficacy of subcutaneous inclisiran in patients at high cardiovascular risk and with elevated low-density lipoprotein cholesterol. The study found that inclisiran significantly decreased LDL cholesterol levels compared to a placebo. After a single dose of inclisiran, the least-squares mean reduction was 27.9–41.9%, and after two doses, it was 35.5–52.6% [48].

The safety and effectiveness of inclisiran were studied by Wright et al. [49] in individuals with varying degrees of renal impairment (RI). The study indicated that there were no discernible differences in the safety and pharmacodynamic effects of inclisiran in individuals with normal renal function when compared to those with mild, moderate, or severe RI. Inclisiran was found to significantly reduce PCSK9 levels and LDL-C in all renal function groups compared to a placebo (P < 0.001 vs. placebo in all groups). These findings indicate that inclisiran can be safely used in patients with RI without requiring any dose adjustments [49].

The phase III ORION-9 study evaluated inclisiran for the treatment of heterozygous familial hypercholesterolemia. A total of 482 adult patients participated in the study and were randomly assigned to receive subcutaneous injections of inclisiran sodium (300 mg) or placebo on days 1, 90, 270, and 450. By day 510, the research revealed that the inclisiran group had significantly lower LDL cholesterol levels than the placebo group, with percent changes of −39.7% and 8.2%, respectively, and a between-group difference of −47.9% points (P < 0.001). Inclisiran was effective in lowering LDL levels in all genotypes of FH, according to the results [9].

#### 3.3.4 The use of siRNA in ASCVD and ASCVD risk equivalent

A study by Ray et al. (ORION-10) [50] demonstrated the effectiveness of inclisiran in lowering LDL cholesterol in patients with ASCVD. The study showed a significant reduction in LDL cholesterol levels compared to placebo with a mean placebo-corrected percentage change in LDL cholesterol levels of −55.5% on day 90, -52.3% on day 510, and -54.9% on day 540 (P < 0.0001 for all). Nevertheless, adverse events at the injection site were more common in the inclisiran group than the placebo group, with a rate of 2.6% vs. 0.9%, respectively. All of these adverse events were mild and none were severe [50,51].

Inclisiran was studied by Raal et al. for safety and efficacy in South African patients who were at high risk of getting cardiovascular illnesses [9]. The data showed that inclisiran reduced LDL cholesterol levels in the South African patients while being both safe and effective. Inclisiran lowered LDL cholesterol levels by 54.2% compared to placebo, and the corresponding time-averaged reductions were 52.8% (P < 0.0001 for both) [9].

In the ORION-11 phase III study, inclisiran was found to have significant and sustained effects in reducing atherogenic lipoprotein concentrations in patients with ASCVD risk equivalent. The study involved 203 participants without a previous history of ASCVD, but with either type-2 diabetes mellitus, FH, or an expected 10-year risk of ≥20% for CVD. The participants were randomized to be given either a placebo or 300 mg of inclisiran sodium at specific intervals over 18 months. The percentage change in the concentration of LDL cholesterol from baseline was −41.9% with inclisiran compared to +1.8% with the placebo, resulting in a −43.7% difference between the groups (95% CI, −52.8 to −34.6; significant p-value) on day 510. Additionally, inclisiran was observed to significantly reduce non-HDL cholesterol and apoB compared to the placebo on day 510 [52]. A phase III pooled analysis of ORION-9, ORION-10, and ORION-11 examined inclisiran's potential benefits in reducing Major Adverse Cardiovascular Events (MACE). The MACE, including fatal and non-fatal incidences of myocardial infarctions and strokes, was lower in patients who received inclisiran than those who received placebo. Inclisiran significantly reduced composite MACE (OR 0.74, 95% CI 0.58–0.94), but not fatal and non-fatal myocardial infarctions (OR 0.80, 95% CI 0.50–1.27) or fatal and non-fatal strokes (OR 0.86, 95% CI 0.41–1.81) [51].

#### 3.3.5 The use of siRNA in hATTR amyloidosis

Mutations in the transthyretin (TTR) gene lead to multisystem organ dysfunction in the rare and progressive condition known as hereditary transthyretin-mediated (hATTR) amyloidosis. Pathogenic TTR aggregation, misfolding, and fibrillization are the causes of amyloid buildup in numerous organs, including the peripheral nervous system and the heart. Our systematic literature search identified two siRNA treatments, Revusiran and patisiran, that are being investigated as potential therapies for hATTR amyloidosis [53,54].

Revusiran is a form of siRNA that specifically targets a section of the human TTR mRNA and is delivered to the liver, where TTR is mostly generated, by a triantennary N-acetylgalactosamine (GalNAc) ligand. Revusiran was well tolerated in phase I and phase II clinical trials and decreased serum transthyretin levels by up to 92.4%. This prompted the phase III ENDEAVOUR study, in which patients with hATTR amyloidosis and cardiomyopathy were enrolled. They were randomized in a 2:1 ratio to receive subcutaneous revusiran 500 mg (n = 140) or placebo (n = 66) daily for 5 days over the course of a week, followed by weekly doses [55].

In the revusiran phase III ENDEAVOUR study, there were more deaths in the revusiran group (18 out of 140 subjects, 12.9%) than in the placebo group (2 out of 66 subjects, 3.0%). The cause of the increased mortality associated with revusiran was investigated, but no definitive explanation was found, although revusiran could not be ruled out as a contributing factor. As a result, the study was stopped and further development of revusiran was discontinued [55].

A lipid nanoparticle (LNP) that makes it easier for the short interfering RNA to be transported to the hepatocytes, the main site of TTR, is included in patisiran, a type of RNAi therapy. By cleaving TTR messenger RNA once inside the cell, the small interfering RNA lowers the production of both mutant and wild-type TTR protein [56].

A phase I clinical study of patisiran by Coelho et al. [56] demonstrated proof of concept for RNAi therapy targeting messenger RNA. In a phase II open-label extension study in 2020, the long-term safety and tolerability of patisiran were assessed. One patient withdrew after roughly 19 months owing to gastro-esophageal cancer (which was assessed unlikely to be related to patisiran), and one patient died of myocardial infarction (not related to patisiran) after completing all doses but before the research's end. Of the 27 individuals who were initially enrolled, 25 patients finished the study. The most frequent drug-related side effects were mild flushing and infusion-related reactions, which occurred in 16 (59%) patients, and no patient discontinued the study as a result.

The effect of patisiran 0.3 mg/kg on individuals with polyneuropathy due to hATTR amyloidosis was examined in the APOLLO phase III trial. There were 225 patients total, 148 of whom received patisiran, and 77 who received a placebo. The modified Neuropathy Impairment Score+7 (mNIS+7) was changed from baseline after 18 months of treatment, according to the study. The results showed that the patisiran group had a decrease of −6.0 ± 1.7 points, indicating less impairment, while the placebo group had an increase of +28.0 ± 2.6 points, indicating more impairment (mean difference, −34.0 points; P < 0.001) [57].

A study by Obici et al. aimed to determine the effect of patisiran on the Quality of Life (QOL) of patients with hATTR amyloidosis. Many assessments were employed in the study, including the Norfolk Quality of Life-Diabetic Neuropathy (Norfolk QOL-DN), EuroQoL 5-dimensions 5-levels (EQ-5D-5L), EQ-VAS, Rasch-built Overall Disability Scale (R-ODS), and Composite Autonomic Symptom Score-31 (COMPASS-31). The results of the study are presented in a table and show that patients who received patisiran had better QOL across all measures compared to those who received placebo [58].

The efficacy of patisiran on a specific cardiac subpopulation from the APOLLO study participants was further analyzed by Minsamisawa et al. [59] and Solomon et al. [60]. Of 256 eligible patients, 126 (56%) did not have hypertension or aortic valve disease and had a baseline LV wall thickness of less than 13 mm. At 18 months, several cardiac parameters were observed and compared to placebo. In the cardiac subpopulation, it was discovered that patisiran dramatically reduced mean LV wall thickness, increased end-diastolic volume, decreased brain natriuretic peptide's N-terminal prohormone, and reduced overall longitudinal strain. These results imply that patisiran may slow or stop the progression of hATTR amyloidosis' cardiac symptoms.

## 4 Conclusion

RNA-based therapy has shown promising results in the management of CVDs and CV risk factors. However, data are still at an early stage and more studies are needed to address several challenges regarding the use of RNA-based therapy in clinical settings. Overall, either RNA-based therapy potentially revolutionizes cardiovascular disease therapy or provides a new avenue as an alternative therapeutic approach in the future.

## List of Abbreviations

ANGPTL3: Angiopoietin-Like 3
apo[a]: apolipoprotein(a)
apoA: Apolipoprotein A
apoB: Apolipoprotein B
apoC: Apolipoprotein C
apoE: Apolipoprotein E
ASCVD: atherosclerotic cardiovascular disease
ASO: antisense oligonucleotide
ATTR: amyloidosis transthyretin protein
CAD: Coronary artery disease
CI: confidence interval
COMPASS-31: Composite Autonomic Symptom Score-31
CRP: C-reactive protein
CV: cardiovascular
CVD: cardiovascular disease
DNA: Deoxyribonucleic Acid
dsRNA: double-stranded RNA
EQ-5D-5L: EuroQoL 5-dimensions 5-levels
EQ-VAS: EuroQoL Visual analogue scales
FH: Familial hypercholesterolemia
FIXa: Factor IXa
FXI-ASO: Factor XI Antisense Oligonucleotide
GalNAc: N-acetyl-galactosamine
GalNAC3: triantennary N-acetylgalactosamine
GPIb-vWF: glycoprotein Ib-von Willebrand factor
HATTR: hereditary transthyretin-mediated amyloidosis
HDL: high-density lipoprotein
IDL-C: Intermediate-density lipoproteins
IQR: interquartile range
LDL-C: low-density lipoprotein cholesterol
LNP: lipid nanoparticle
Lp(a): Lipoprotein(a)
LPL: Lipoprotein lipase
LV: Left Ventricle
MACE: Major Adverse Cardiovascular Events
miRNA: microRNA
mNIS+7: Neuropathy Impairment Score+7
MRI: Magnetic Resonance Imaging
mRNA: messenger RNA
nc-RNA: non-coding RNA
Norfolk QOL-DN: Norfolk Quality of Life-Diabetic Neuropathy
OR: Odds Ratio
PAC-1: Procaspase Activating Compound-1
PAD: peripheral arterial disease
PCI: Percutaneous Coronary Intervention
PCSK9: Proprotein convertase subtilisin type 9
PICO: Population, Intervention, Comparison and Outcomes
post-SARS-CoV-2: post Severe acute respiratory syndrome coronavirus 2
PPM: permanent pacemakers
P-selectin: Platelet-selectin
QOL: Quality of Life
RCT: randomized controlled trials
RI: renal impairment
RISC: : RNA-induced silencing complex
RNA: Ribonucleic Acid
RNase H: Ribonuclease H
RNAi: RNA interference
RoB: risk-of-bias
ROBINS-I: Risk Of Bias In Non-Randomized Studies of Interventions
Robvis: Risk-of-Bias visualization
R-ODS: Rasch-built Overall Disability Scale
SELEX: systemic evolutions of ligands by exponential enrichment
siRNA: small-interfering RNA
T2DM: type 2 diabetes mellitus
TTR: transthyretin protein
VLDL: very-low-density lipoprotein
vWF: von Willebrand Factor
