# Supplementary material for: Non-coding RNA therapeutics in cardiovascular diseases and risk factors: Systematic review
Source: Noncoding RNA Res. 2023 Jul 1;8(4):487–506. doi: 10.1016/j.ncrna.2023.06.002 (PMC10362275; doi:10.1016/j.ncrna.2023.06.002)
Supplement: Multimedia component 1 [file mmc1.docx]

**Supplementary material 1 : Research keywords**

*Pubmed*

((RNA[Title/Abstract]) OR (miRNA[Title/Abstract]) OR (microRNA[Title/Abstract]) OR (microRNA[MeSH Terms] OR (aptamer[Title/Abstract]) OR (siRNA[MeSH Terms]) OR (siRNA[Title/Abstract]) OR (aptamer[Title/Abstract]) OR (aptamer[MeSH Terms]) OR (aso*[Title/Abstract]) ) OR (antisense oligonucleotide[Title/Abstract])) AND ((therapy[Title/Abstract]) OR (therapeutic*[Title/Abstract]) OR (management[Title/Abstract])) ) AND ((cardiovascular disease*[Title/Abstract]) OR (cardiovascular diseases[MeSH Terms] ) OR (heart failure[Title/Abstract]) OR (HF[Title/Abstract]) OR (myocardial disease*[Title/Abstract]) OR (reperfusion injury[Title/Abstract]) OR (arrhythmia[Title/Abstract]) OR (cardiac conduction disorder[Title/Abstract]) OR (dyslipidemia[Title/Abstract]) OR (diabetes[Title/Abstract]) OR (DM[Title/Abstract]) OR (Hypertension[Title/Abstract]) OR (HT[Title/Abstract]) OR (PAD[Title/Abstract]) OR (peripheral artery disease*[Title/Abstract]) OR (embolism[Title/Abstract]) OR (amyloidosis[Title/Abstract]) OR (cardiomyopathy[Title/Abstract]) OR (ischemic[Title/Abstract]) OR (ischemic heart disease*[Title/Abstract]) OR (cardiovascular risk factor*[Title/Abstract]))

AND

(non coding rna[MeSH Terms]) AND (cardiovascular diseases[MeSH Terms]) AND (clinicaltrial[Filter]) AND (Therapy) Filters: Clinical Trial, Randomized Controlled Trial

("rna, untranslated"[MeSH Terms] AND "cardiovascular diseases"[MeSH Terms] AND "clinical trial"[Publication Type] AND ("therapeutics"[MeSH Terms] OR "therapeutics"[All Fields] OR "therapies"[All Fields] OR "therapy"[MeSH Subheading] OR "therapy"[All Fields] OR "therapy s"[All Fields] OR "therapys"[All Fields])) AND (clinicaltrial[Filter] OR randomizedcontrolledtrial[Filter])

AND

(("rna"[MeSH Terms] OR "rna"[All Fields]) AND ("therapeutics"[MeSH Terms] OR "therapeutics"[All Fields] OR "therapies"[All Fields] OR "therapy"[MeSH Subheading] OR "therapy"[All Fields] OR "therapy s"[All Fields] OR "therapys"[All Fields]) AND ("heart diseases"[MeSH Terms] OR ("heart"[All Fields] AND "diseases"[All Fields]) OR "heart diseases"[All Fields] OR ("cardiac"[All Fields] AND "disease"[All Fields]) OR "cardiac disease"[All Fields])) AND (clinicaltrial[Filter])

FILTER: Full text, English

*Cochrane*

#1 MeSH descriptor: [Cardiovascular Diseases] explode all trees 119552

#2 (cardiovascular disease*):ti,ab,kw OR (heart NEXT (disorder OR disease)):ti,ab,kw OR (cardiovascular risk factor*):ti,ab,kw OR ((cardiac OR vascular) NEXT (disorder* OR disease*)):ti,ab,kw (Word variations have been searched) 81891

#3 #1 OR #2 173705

#4 MeSH descriptor: [RNA] explode all trees 4178

#5 (non-coding RNA):ti,ab,kw OR (microRNA):ti,ab,kw OR (siRNA):ti,ab,kw OR (aptamer):ti,ab,kw OR (antisense oligonucleotide*):ti,ab,kw (Word variations have been searched) 1902

#6 #4 OR #5 5772

#7 #3 AND #6 539

#8 MeSH descriptor: [Therapeutics] explode all trees 331621

#9 (therapy*):ti,ab,kw OR (management):ti,ab,kw (Word variations have been searched) 861658

#10 #8 OR #9 951586

#11 #7 and #10 391

*Scopus*

*( TITLE-ABS-KEY ( rna ) AND TITLE-ABS-KEY ( cardiovascular AND diseases ) AND TITLE-ABS-KEY ( therapy ) ) AND ( LIMIT-TO ( PUBSTAGE , "final" ) ) AND ( LIMIT-TO ( DOCTYPE , "ar" ) ) AND ( LIMIT-TO ( LANGUAGE , "English" ) ) AND ( LIMIT-TO ( SUBJAREA , "MEDI" ) OR LIMIT-TO ( SUBJAREA , "BIOC" ) OR LIMIT-TO ( SUBJAREA , "PHAR" ) ) AND ( LIMIT-TO ( SRCTYPE , "j" ) )*

*Directory of Open Access Journal (DOAJ)*

'non coding RNA' and 'Cardiovascular Disease'

**Supplementary material 2: Included Studies**

| Study registration number (ref) | Study Population | Age Group | Intervention Arm | n | Comparator arm | n | Time to follow Up | Primary Endpoint | Outcome: Mean Difference (Treatment vs Placebo) | Outcome: Change from Baseline (95% CI) | | Adverse Events |
| --- | --- | --- | --- | --- | --- | --- | --- | --- | --- | --- | --- | --- |
|  |  |  |  |  |  |  |  |  |  | Placebo | Study Drug |  |
| Aptamers | | | | | | | | | | | | |
| Povsic et al. 2013 | Non-ST-elevation ACS patients with planned early cardiac catheterization via femoral access <24h. Past medical history of CHF, MI, Previous PCI, Previous CABG, HTN, T2DM, Renal Insuficiency, Stroke, Current tobacco use. | 25 to 75 | Pegnivacogin 1mg/kg and Anivamersen reversal 0.075, 0.20, 0.40, 1.00 mg/kg | REG1 25 to 100% (n= 40,113,119,194) | Heparin | (n=161) | 30 days | Primary endpoint: total ACUITY bleeding. Secondary endpoints: major bleeding and ischaemic event. | Total bleeding (%) 33.7, 2.7, 3.7, -1.3;  Major bleeding (%) 10, 1, -2, -3;  Ischaemic event (%) -2.7 (0.2,1.4) | Total bleeding 31.3%. Major bleeding 10%. Ischaemic event 5.7%. Incidence of the composite ischaemic endpoint (n=9) (death (n=1), non-fatal myocardial infarction (n=7), urgent target vessel revascularization (n=1), or recurrent ischaemia in the target vessel distribution) through a 30-day follow-up of patient with Heparin. | Total bleeding 65, 34, 35, 30 (p=0.1 when discharge and 0.9 in 30-day follow up); major bleeding 20, 11, 8, 7 ; ischaemic event 3.0 (ischaemic event 95% CI = 0.2-1.4) in REG1. Incidence of the composite ischaemic endpoint REG1 (n=3, 1, 5, 5) (death (n= 0, 0, 1, 0) , non-fatal myocardial infarction (n= 3, 1, 4, 4), urgent target vessel revascularization, or recurrent ischaemia in the target vessel distribution (n= 1, 0, 1, 1)) through a 30-day follow-up | REG1 n=60. Heparin n=55. 3 incidence of allergic-like adverse events within 24 h of drug administration, 2 of 3 are SAE. REG 1 (hives 0.2%, hypotension 2.4%, rash 0%, dyspnoea 0.9%). Heparin (hives 0%, hypotension 1.9%, rash 0.7%, dyspnoea 0%) |
| Arzamendi et al. 2011 | CAD patient on double antiplatelet therapy and normal volunteers (CAD patient n=27 (Male (n=22), Hypertension (n=9), Hypercholesterolemia (n=16), T2DM (n=4), Smoker (n=10), ACS (STEMI n=17; NSTEMI n=1; UA=9)), Healthy volunteers n=5) | 18 to 75 | ex vivo treated pretherapy (incubated 5 minutes before the onset of perfusion) or 10 min post therapy on damage arteries with: ARC1779 (25, 83, and 250 nmol/L), or Abciximab (100 nmol/L), or placebo | n=27 | placebo | n | 15 min | Platelet function |  | Pretherapy with placebo in healthy patients (n=5) effect on platelet adhesion: 81.9 ± 23.6 × 10^6 platelets/cm2 |  |  |
| Staudacher et al. 2019 | Healthy volunteers and patient with ACS | ≥ 18 (whole blood sample) | Pegnivacogin or Pegnivacogin 1 mg/kg +Anivamersen (RNA Aptamer reversal agent) | n | Placebo | n | 20 min | CD62P-expression, PAC-1 binding | Pegnivacogin vs placebo  CD62P expression  20 mikroM ADP (n=9): -13.38 p=0.027  1 mikroM ADP (n=24): -6.59 p=0.031  PACbinding  20 mikroM ADP (n=11): -16.98 p=0.0098  1 mikroM ADP (n= 25): -9.59 p=0.0008  20 mikroM ADP (n=10): -2.42 p=0.922  1 mikroM ADP (n=3): -2.38 p=0.449  Blood from healthy subject after ex-vivo incubation with 150 µl pegnivacogin: platelet aggregation -3.66% p=0.002, n=10  Patient CAD treated dual antiplatelet after 20min iv 1 mg/kg pegnivacogin: platelet aggregation -56.79% p=0.020, n=3 | | |  |
| Chan et al. 2008 | subjects with stable CAD | 50 -75 | aptamer (RB006) sd 1 min iv  ASO (RB007) sd 3h iv | Group 1 = 28;  Group 2 (+ placebo antidote) = 14 | placebo | 8 | day 7 | safety, tolerability, pharmacodynamic | RB006 increased the activated partial thromboplastin time dose dependently; the median activated partial thromboplastin time at 10 minutes after a single intravenous bolus of 15, 30, 50, and 75 mg RB006 was 29.2, 34.6, 46.9, and 52.2 seconds, P<0.0001. RB007 reversed the activated partial thromboplastin time to baseline levels within a median of 1 minute with no rebound increase through 7 days. | | |  |
| Cohen et al. 2010 | undergo non-urgent PCI  have a prior indication for PCI  pre-treatment with aspirin and clopidrogel | 18-80 adult | RB006 1 mg/kg / IV, SD  RB007 0.2:1 (50% efficacy)/ 2:1 (100%), SD | 20 | UFH IV treated | 4 | 48 hours  14 days | major bleeding 48 hrs/ hospital discharge  all-cause death, MI- events, urgent revasc 14 days | 1.0 Median (0.9, 1,1) p< 0.001 |  |  | A total of 4 AEs, 2 patients from treatment group  2 patients from control/comparison group |
| Antisense Oligonucleotides (ASOs) | | | | | | | | | | | | |
| Furtado et al. 2012 | hypercholesterolemic LDL-C ≥130mg/dL and TG≤4oomg/dL  BMI 25-32kg/m2 | 18 to 65 yo | Mipomersen once a week.  Doses  A: 100 mg  B: 200 mg  C: 300 mg  For a total of 13 weeks | A:8  B:8  C:6(apoCIII) | Placebo | 2  2  2 | Day 99 | Total cholesterol;  Concentration of ApoCIII;  concentration of apoB | Total Cholesterol  100 mg = -33.3 (-60.6, -5.9) p=0.004  200 mg = -78.4 (-105.7, -51.1) p<0.001  300 mg = -108.5 (-136.9, -81.0) p<0.001  ApoB  100 mg = -30.3 (-46.2, -14.3) p=0.001  200 mg = -57.3 (-72.3, -42.3) p<0.001  300 mg = -84.5 ( -100.3, -68.7) p<0.001  ApoCIII  100 mg = 0.97 (-3.1, 5.1) p=0.6  200 mg = -5.81 (-10.1, -1.5) p=0.01  300 mg = -6.03 (-8.9, -3.2) p<0.001 | | | Not reported |
| Viney et al. 2016 | 64 participants to the phase 2 trial (35 in IONIS-APO(a)Rx and 29 in placebo in June 25, 2014, to Nov 18, 2015). 58 healthy volunteers to the phase 1/2a trial of IONIS-APO(a)-LRx (28 in sd group and 30 in md group in April 15, 2015, to Jan 11, 2016) | Adult | A: IONIS-APO(a)Rx 100 mg SC, once a week for 4 weeks, 200 mg SC, once a week for 4 weeks, then 300 mg SC, once a week for 4 weeks  B: IONIS-APO(a)-LRx 6 doses of 10 mg, 20 mg, or 40 mg at days 1, 3, 5, 8, 15, and 22, for a total dose exposure in the active arms of 60 mg, 120 mg, or 240 mg per cohort. | A: 51  B: 13 | Placebo | A: 26  B: 3 | A: day 85 or 99  B: day 30 | A: reduction of Lp(a) plasma concentration  B: reduction of Lp(a) plasma concentration | A: 66.8% (61.6, 72)  B: 24·8% (3·1, 67·1) for  10 mg,  35·1% (2·2, 8·8) for 20 mg,  48·2% (10·9, 78·4)  for 40 mg,  82·5% (50·5, 109·2) for 80 mg,  84·5%  (65·2, 112·6) for 120 mg |  |  | There were two serious adverse events (myocardial infarctions) in the IONIS-APO(a)Rx phase 2 trial, one in the IONIS-APO(a)Rx and one in the placebo group, but neither were thought to be treatment related. 12% of injections with IONIS-APO(a)Rx were associated with injection-site reactions. IONIS-APO(a)-LRx was associated with no injection-site reactions. |
| Tsimikas et al. 2015 | healthy adults, BMI less than 32•0 kg/m(2), Lp(a) 25 nmol/L (100 mg/L) or more | 18-65 years | ISIS-APO(a)Rx, Single dose, SC injection  A: 50 mg  B: 100 mg  C: 200 mg  D; 400 mg  ISIS-APO(a)Rx, Multi dose, SC injection  A: 100 mg for a total dose exposure of 600 mg  B: 200 mg for a total dose exposure of 1200 mg  C: 300 mg for a total dose exposure of 1800 mg | I  A:3  B:3  C:3  D:3  II  A:8  B:8  C:8 | Placebo | Single dose: 4  Multi dose: 6 | day 30  day 36 | Lp(A) reduction | Single doses of ISIS-APO(a)Rx (50–400 mg) did not decrease Lp(a) concentrations at day 30. | 5% (-8,15) | Multidose ISIS-APO(a)Rx  100 mg 39.6% , p=0.005  200 mg 59.0%, p=0.001  300 mg 77.8%, p=0.001 | Mild injection site reactions were the most common adverse events. 2 volunteers excluded due to AE (one each in ISIS-APO(a)Rx 200 mg md (ec injection site adverse event) and 300 mg md (ec flu-like syndrome that resolved without sequelae). No SAE. Mild injection site reactions were the most common adverse events. ≥10% of participant in ISIS-APO(a)Rx group got headache and fatigue, no significantly different compared to Placebo. |
| Waldman et al. 2017 | Established atherosclerosis, LDL >= 3.4 mmol/L (130 mg/dL) despite on stable maximal possible lipid lowering therapy for more than >= 3 months, BMI <= 40 kg/m2, women had to be postmenopausal or on highly effective contraceptive regimen, and fulfilled German criteria for lipoprotein apheresis | > 18 yo, mean 42-72 yo | Mipomersen 200 mg, SC injection, weekly for 26 weeks (at least 12 weaks) | 11 | none | 4 | 26 weeks, or between 12-26 weeks for discontinued patients (n=4) | pre apheresis LDL cholesterol | -0.02 (-1.1, 1.1) p=0.002 | -1.6 (-10.7, 7.51) | -22.6 (-32.6, -12.6) | Of the 11 patients randomized to mipomersen, 3 discontinued the drug early due to side effects (2 for injection site reactions and 1 for ﬂu-like symptoms) and were replaced. Further 4 patients discontinued mipomersen during treatment weeks 12e26, again for side effects (1 due to elevations of liver enzymes, the other 3 due to moderate to rather severe injection site reactions (ISR) and ﬂu-like symptoms (FLS)) and were not replaced. |
| Büller et al. 2015 | undergoing elective primary unilateral total knee arthroplasty | 18 to 80 yo | FXI-ASO, SC injection  200 mg  300 mg  9 times at day 1,3,5,8,15,22,29,36,39 | 134  71 | enoxaparin 40mg | 69 | 3 months | Incidence of adjudicated total thromboembolism which was a composite of asymptomatic DVT, objectively confirmed symptomatic venous thromboemolism, fatal PE, unexplained death which PE could not be ruled out. | Efficacy (Total Venous Thromboembolism)  200 mg = -15 (-37, 7) p=0.59  300 mg = -18 (-16, -29) p<0.001 |  |  | Total 12 AE (bleeding), 6 of which related to treatment. |
| Dasgupta et al. 2020 | Biopsy proven ATTR amyloidosis (hereditary or wild type) with clinical signs and symptoms of CHF (NYHA I - III), a left ventricular wall thickness >= 1.3 cm on TEE, stable renal function (GFR > 35) and stable thyroid function (TSH < 10 or normal serum T4) | No age restriction (mean 63.4 - 76.2 yo) | Inotersen  300 mg/1.5 ml subcutaneous/week | 33 | None | None | Every 6 months, published) | Decrease of LV mass (MRI), Decrease in left ventricular septal thickness (TEE), Increase of exercise tolerance (6MWT), Stable LVEF, Steady decline of BNP. |  |  |  | Total 8 AE, all related to treatment. AEs:  Inflammation & Induration on the site of injection |
| Tsimikas et al. 2020 | elevated screening plasma lipoprotein(a) level (≥60 mg per deciliter [150 nmol per liter]).  Confounding factors: CAD, Overweigh, HT, DMT2, Familial Hypercholesterolemia, smoking | Adult >18-80 | APO(a)-LRx, SC injection  20 mg every 4 weeks,  40 mg every 4 weeks,  60 mg every 4 weeks,  20 mg every 2 weeks  for 6 months | 48,  48,  47,  48,  48 | Placebo normal saline per week | 47 | 6 months | percent change in Lipo(a) at 6 monts exposure, safety and efficacy | 20 mg/4 weeks = 80.7 (1.2, 21) p=0.003 ;  40 mg/4 weeks = 101.7 (7.3, 131.4) p<0.001;  20 mg/2 weeks = 115.1 (9.8, 195) p<0.001;  60 mg/ 4 weeks = 134.3 (24, 627.4) p<0.001;  20 mg/1 weeks = 172.6 (109.3, 11.571)p<0.001 |  |  | Total 253 AE, 212 of which related to treatment.  2 deaths due to traffic accident and suicide.  AEs:. influenza like symptoms, injection site reaction |
| Santos et al. 2015 | HoFH, Severe-HC, HeFH-CAD, HC-CHD | >= 12 y | Mipomersen 200 mg, SC injection, weekly for 26 weeks | Total 261  HoFH 51,  Severe-HC 58,  HeFH-CAD 124,  HC-CHD 157 | placebo | 129 | week 28 - week 28+24 | LDL-C | HoFH = 0.3 (0.04, 0.6), p=0.002 ;  Severe-HC = 0.6 (0.4, 0.8) p=0.002 ;  HeFH-CAD = 0 (-0.2, 0.2) p=0.001 ;  HC-CHD = 0.6 (0.4, 0.8) p<0.001 |  |  | injection site reaction (+).  N= ?? |
| Santos et al. 2015 | HoFH, Severe-HC, HeFH-CAD, HC-CHD  Comorbidites: smoker, metabolic syndrome, overweight-obese |  | Mipomersen 200 mg, SC injection, weekly for 26 weeks | 382  HoFH 51,  Severe-HC 57,  HeFH-CAD123,  HC-CHD 151, | placebo | 126 | week 28 | Lp(a) | -26.4 (-32.1, -20.7) p<0.001  median (interquartile range) |  |  |  |
| Thomas et al. 2013 | HC, CHD  Comorbidities: DMT2 | >=18 | Mipomersen 200 mg SC injection weekly, for 28 weeks | 101 | placebo | 50 | week 28 - week 24 | LDL-C | -38 (-49.3824, -26.6176) p<0.001 | −4.5 ± 24.22 | −36.9 ± 26.85 | A total of 139 patietns experiencing AEs, 97 of which related to treatment.  AEs: injectio site reaction, flu-like symptoms, ALD increased, hepatic stetosis |
| Luigetti et al. 2022 | hereditary aTTR |  | inotersen 14.6 ± 5.9 months (range, 6–24 months) | 23 | none | none | 6 to 14.6 months | troponin, NTpro BNP, intervent septum thickness, BMI  safety --> number of dropouts | Troponin 0.01 (-0.0052, 0.0252) p=0.19 ;  NTpro BNP -45.6 (-703.82, 612.62) p=0.88  IVS 1.5 (-0.46, 3.46) p=0.12 |  |  | 5 dropouts, 2 of which related to treatment.  20 AEs are all related to treatment, which are:  4: severe thrombocytopenia  9: mid trombocytopenia  7: mild thrombocytopenia |
| [Yang et](http://et.al/) al. 2016 | hyperTAG  cohort 1: FCS  cohort 2: hyperTAG of varying causes  cohort 3: stable fibrate therapy | adult | Volanesorsen  100 mg,  200 mg,  300 mg  weekly for 13 weeks | 11,  13,  11 | Placebo | 16 | 176 days | apoCIII-apoB | apoCIII-ApoB 100 mg -31 (-17005, 16943)  apoCIII-ApoB 200 mg 21026 (8505, 33547) p <0.001  apoCIII-ApoB 300 mg -626803 (-640678, -612928) p <0.001 |  |  | not reported |
| Benson et al. 2017 | hereditary and wild-type ATTR with moderate-severe cardiomyopathy  biopsy-proven | adult/elderly >55 years | IONIS-TTR]) |  |  |  |  |  |  |  |  | 10 patients experiencing AEs |
| Reeskamp et al. 2018 | high risk and severe HeFH  persistent hyperchol  maximal LDL-lowering therapy  Comorbidities:  Smoking  Alcohol consumption  HoFH  CHD  other atherosclerotic disease  Hypertension  DM  statin | Adult>18 yo | Mimopersen  200 mg SQ 1x/week  70 mg SQ 3x/week  for 60 weeks | 133 --> 104  73 --> 102 | Placebo | 67 --> 57  38 --> 25 | 84 weeks | percent change LDL | -20.96 (-29.5085, -12.4115) p<0.001  -18.80 (-20.7270, -16.8730) p<0.001 |  |  | A total of 259 AEs, 178 of which related to treatment |
| Sugihara et al. 2015 | dual chamber PPM and AF burden 1-10  comorbidities:  Use of dabigatran/warfarin  HT  DM  Hyperlipidemia  Hypothyroidism  Prior stroke  Use of card meds as indicated above | adult >18 yrs | ISIS-CRPRx  200 mg in 1 mL solution/SC in two injection  3x/wk for 1 week  1x/wk for 3 wks  Total intervention: 4 weeks | 7 | none | none | every visit during drug administration;  4 week; and  8 week | change in AF burden before and after |  |  | MD: 1.6% (-1.45% to 4.65%) p=0.37  CRP: -2.9 (-5.95, 0.15) mg/L p=0.031 |  |
| Viney et al. 2021 | healthy, non-pregnant,/lactating, BMI <32, able to take vitamin A | 18-65 adults | AKCEA-TTR-LRx (ION-682884)  120 mg SD/SC  45 mg 4x dose/SC, 1x/month for 4 months  60 mg 4x dose/SC, 1x/month for 4 months  90 mg 4x dose/SC, 1x/month for 4 months | 9  10  10  10 | placebo | 2  2  2  2 |  | safety assesment : AEs --> physical and lab findings  PK parameters  PD parameters | TTR  SD: -80.40 (-94.0 to -66.8)  45 mg : -79.80 (-95 to -64.6), p<0.001  60 mg: -84.60 (-98.9 to -70.3), p<0.001  90 mg: -87.90 (-97.4 to -78.4), p<0.001 |  |  | A total of 7 AEs, 6 of which related to treatment. |
| Benson et al. 2018 | stage 1 and 2 hereditary TTR amyloidosis  comorbidities:  Val30Met TTR mutation  stage 1 vs stage 2  Previous treatment with tafamidis and diflusinal | adults | inotersen 300 mg, SC injection, 3 injection for the 1st week, followed by weekly injection up to 65 wks (67 doses) | 87 | placebo | 52 | 1 week after initiation  35 wks after initiation  66 week post treatment | "mNIS+7 score  Norfolk QOLD-DN score" | mNIS+7: -19.70 (-21.3 to -18.1) p<0.001  norfolk QOL-DN: -.17( xxxxx) p<0.001 |  |  | A total of 199 AEs, 119 of which related to treatment.  110 --> any AEs  9 --> serious AEs. |
| sIRNA | | | | | | | | | | | | |
| INCLISIRAN | | | | | | | | | | | | |
| Fitzgerald et al. 2014 | Healthy adults with LDL-C higher than 3.00mmol/L | 18-65 | ALN-PCS one dose IV  0.015 mg/kg  0.045 mg/kg  0.090 mg/kg  0.150 mg/kg  0.250 mg/kg  0.400 mg/kg | Total 24  3  3  3  3  6  6 | Placebo (NS) | 8 | Data for adverse event : 28 days  Other data : 180 days | Safety, tolerability, and adverse event | Mean percentage change vs placebo: PCSK9 (-45.3 , -86.0, -71.5, -96.2, -98.3, -114.5); LDL-C (-6.6 , -13.4 , -27.2 , -24.0 , -30.1 , -47,2) | PCSK9 change from baseline (-8.7%); LDL-C (-24.0%) | PCSK9 change from baseline (-30.8 , -52.9 , -45.9, -64.2, -58.5, -58.6%); LDL-C (-14.4 , -19.3 , -30.4 , -35.0 , -35.5 , -36.1%) | Treatment-emergent adverse events (TEAE)  (rash, headache, hiccups, cold symptoms, paraesthesia, polyuria or dysuria, infusion-site hematoma)  ALN-PCS n=19 (79%)  Placebo n=7 (88%) |
| Fitzgerald et al. 2017 | Healthy volunteers with LDL cholesterol level >=100 mg/dl, TG level <=400 mg/dl | 18 to 65 yo sd phase,  18 to 75 yo md phase | Single dose phase: sc inclisiran (n=4 each)  25 mg  100 mg  300 mg  500 mg  800 mg (two cohorts for the 800-mg dose).  Multi-dose phase: (n=4-8 each)  125mg/w for 4 weeks,  250mg/2 weeks for 4 weeks,  300mg/month for 2 months with and without statin,  500mg/month for 2 months with and without statin | SD 4 each  MD 4-8 each | Placebo | SD n=6,  MD phase n=11 (8 in md phase without statin group and 3 in md phase with statin group) | 56 days for sd phase, ≤84 days for md phase.  PD end point were evaluated for an additional month (until 180 days after last dose of therapy) after completion of safety and side effect profile assessment | Safety, side effect profile | sd phase: PCSK9 (-46.0 ; -31.4 ; -73.9 ; -69.3 ; -72.5)  sd phase: PCSK9 (<0.05 ; n/a ; <0.001 ; <0.001 ; 0.001) | in sd phase: reduce PCSK9 (-0.6%) | sd phase: reduced in PCSK9 (-46.6 ; -32.0 ; -74.5 ; -69.9 ; -73.1) | No SAE, most common adverse events were cough, musculoskeletal pain, nasopharyngitis, headache, back pain, and diarrhea.  in sd phase (≥5% participant in inclisiran group): 2 of 18 cough, musculoskeletal pain, nasopharyngitis. In md phase (≥10% participant in inclisiran group) 6 of 33 headache, 5 (15%) diarrhea, 5 (15%) back pain, 4 (12%) nasopharyngitis |
| Ray et al. 2017 | LDL > 70mg/dL for patient with history ASCVD/ >100mg/dL without history ASCVD  max statin therapy | 62-74 | SD :  - Placebo  - 200mg  - 300mg  - 500mg  DD :  - Placebo  - 100mg  - 200mg  - 300mg | 370 | Placebo | 127 | Primary 180 days  Other data 210-240 days | Percentage change from baseline in LDL-Cholesterol level |  | SD 2.1  DD 1.8 | Percentage change from baseline LDL - C Levels (Data are Least-squares means)  SD -27.9 ; -38.4 ; -41.9  DD -35.5 ; -44.9 ; -52.6 | Serious Adverse events  Intervention group SD 200mg 6 (10%); 300mg 5 (8%) ; 500mg 6 (9%) ; DD 100mg 11 (18%) ; 6 (10%) ; 7 (11%)  Placebo SD placebo 3 (5%) ; DD placebo 6 (10%) |
| Raal et al. 2020 | Diagnosed with heterozygous familial hypercholesterolemia  LDL at least 100mg/dL despite max statin therapy | 47-64 | Inclisiran SC 300mg  Day 1, 90, 270, 450 | 242 | Placebo | 240 | Day 30, 150, 330, 510, 540 | I = Percentage change from baseline LDL - C levels on day 510  II = time-averaged percent change in the LDL - C level between day 90 and day 540 | I = -47.9% (95% CI, -53.5 to -42.3; P<0.001)  II = -44.3% (95% CI, -48.5 to -40.1; P <0.001) | I = 8.2% (4.3 to 12.2)  II = 6.2% (3.3 to 9.2) | I = -39.7% (-43.7 to -35.7)  II = - 38.1% (-41.1 to -35.1) | Patients with >= 1 serious adverse event  - Intervention group 18 (7.5%) , 1 death from cardiovascular cause 1 (0.4%)  - Control 33 (13.8%) |
| Ray et al. 2022 | high risk, primary prevention patients or those with ASCVD (secondary prevention) | ≥18 | Inclisiran 300mg SD SC  Day 1, 90, 270, 450 | 98 | Placebo | 105 | up to day 540 | Percentage change in LDL-C from baseline at day 510 and time adjusted percentage change in LDL-C from baseline after day 90 and up to day 540 | LDL-C changes from baseline to day 510 (-43.7%). Time adjusted change in LDL-C from baseline after day 90 up to day 540 (-41.0%). Absolute change difference of LDL-C is -.5mmol/dL (-58.4mg/dL) between groups  P <0.0001 , <0.0001 , <0.0001 | The mean percentage changes in LDL-C levels from baseline to day 510 was +1.8% with placebo. Mean baseline LDL-C= 3.6mmol/L. The mean time adjusted percentage change in LDL-C from baseline after day 90 up to day 540 was +0.6% for placebo. The absolute change in LDL-C from baseline to day 510 was -0.06% (-2.3mg/dL) in placebo group. | The mean percentage changes in LDL-C levels from baseline to day 510 was -41.9% with inclisiran. The mean time adjusted percentage change in LDL-C from baseline after day 90 up to day 540 was -40.4% for inclisiran. The absolute change in LDL-C from baseline to day 510 was -.1,6mmol/L (-60.7mg/dL) in inclisiran group | SAE, AE at injection site  Intervention : SAE (n=20), AE at injection site (n=4)  Control : SAE (n=13), AE at injection site (n=0) |
| Wright et al .2021 | ITT Population (For efficacy analyses: Pooled analysis of ORION-9,-10 and -11, included patients with heterozygous familial hypercholesterolaemia, atherosclerotic CV disease (ASCVD), or ASCVD risk equivalent on maximally tolerated statin-therapy  Population (for Safety analyses ) : All patients who received at least 1 dose of Inclisiran / placebo | 54 - 73 | Inclisiran 284mg SD SC  Day 1, 90 and 6-monthly until 18months | 1833 | Placebo | 1827 | 540 | Change in LDL- C Levels & Safety population (risk of cardiovascular events) | Change in LDL-C levels  day 90 :  - 50.6% [95% CI  (−52.3 to −49.0); P<0.0001]  day 540 :  - 51.4% [95% CI (−53.4 to −49.4); P<0.0001]  Inclisiran significantly reduced MACE (OR[95%CI] : 0.74 [0.58-0.94]), but not fatal and non-fatal MI (OR [95% CI] : 0.80 [0.50−1.27]) and fatal and non-fatal stroke (OR [95% CI] : 0.86 [0.41−1.81]). |  |  | - MACE  Intervention : 131  Control :171  - Fatal and non-fatal MI  Intervention : 33  Control :41  - Fatal and non-fatal stroke  Intervention : 13  Control :15 |
| Ray et al. 2020 | ASCVD, LDL >70, statin and lipid lowering therapy use, GFR >30 | adult  >18 years | Inclisiran 284 mg/SC | ORION 10 : 781  ORION 11 : 810 | Placebo | 780  807 | day 30  day 150  day 330  day 510  day 540 | Percentage change in LDL  time adjusted LDL change (throughout the follow-up period) | -52.3 P <0.001  -49.9 P <0.001 | ORION 10: 1.0%  ORION 11: 4% | ORION 10: -51.3  ORION 11: -45.8% | Total AEs : 1156  Serious AEs :  ORION 10: 175  ORION 11: 181 |
| Wright et al. 2020 | Participant with normal renal function and mild, moderate and severe RI from phase 1 ORION -7 renal study and the phase 2 ORION-1 study; BMI 18-40kg/m2 and BW >50kg | 18 < age <80 | (ORION 7) = SD Placebo and 300mg ; DD Placebo and 300mg  (ORION 1 )1 = Normal Function, 2 = Mild RI, 3= Moderate RI, 4= Severe RI | ORION 1 = 122  ORION 7 = 31 | Placebo | ORION 1 = 125  ORION 7 = 0 | 180 days atau max 360 days | ORION 7 : PK, Safety, PD  ORION 1 : PK, Safety |  |  |  | Participants with at least 1 TEAE (treatment-emergent adverse event)  Intervention : 126  Control : 101 |
| Raal et al. 2022 | HeFH  ASCVD ASCVD-risk equivalent | adults | Inclisiran 284 mg/SC | 148 | Placebo | 150 | 510 days | LDL percentage change at day 510  time-averaged percentage LDL change | -54.2% P <0.0001 | 0.077 SD 2.9- 12.6 | -46.5% SD -51.7 - 41.3 | Participants with ≥1 TEAEs : 233  Serious TEAEs :  Intervention: 32  Placebo: 37 |
| PATISIRAN | | | | | | | | | | | | |
| Coelho et al. 2013 | ALLN 01:biopsy-confirmed TTR amyloidosis mild-moderate neuropathy  karnofsky performance status >60  BMI 18.5-33  NYHA II or less  adequate liver, renal, thyroid function  not pregnant/childbearing potential  ALLN 2: 18-45, healthy, BMI 18-31.5. | adults  >18 yr | ALN-TTR01 (0.01 to 1.0 mg/kg) - IV  ALN-TTR02 (0.01 to 0.5 mg/kg) - IV | 24  13 | Placebo | 8  4 | 70 days | reduction in TTR level | -38% (dosage 1.0 mg/kg) P 0.01  ALLN 02  0.15 = -85.7% P 0.001  0.3 = -87.6% P 0.001  0.5:= -93.8% P 0.001 | 0.01  0.001  0.001  0.001 |  | ALN-TTR01  Infusion Reaction : 5  Fatigue : 2  Placebo : 0  ALN-TTR02  Skin Erythema : 6  Infusion Reaction : 1  Placebo : 2 (Skin Erythema) |
| Coelho et al. 2020 | hATTR amyloidosis | 29 to 77 | Patisiran 0.3 mg/kgbb - IV  once / 3 weeks | Patisiran alone (n=7) | Patisiran + TTR tetramer stabilizer | n=19 | 24 months | The primary objective was to evaluate the safety and tolerability of long-term dosing with patisiran. | (-0.28) | (-7.03) SE 2.11 | (-6.75) SE 5.24 | SAE 7, death 2, any AE leading to discontinuation 2. None of which were considered related to Patisiran  flushing (n=7), infusion-related reactions (n=6), diarrhea (n=3) |
| Minamisawa et al. 2019 | Patients with hereditary transthyretin-mediated (hATTR) amyloidosis with polyneuropathy and cardiac amyloidosis | 61 (54-67) | Patisiran 0.3 mg/kgbb - IV  once / 3 weeks | 90 (71.4%) patisiran | Placebo | 36 (28.6%) placebo | 18 months | improved left ventricular (LV) global longitudinal strain (LV GLS) | Patisiran improved the absolute GLS (least-squares mean [SE] difference, 1.4% [0.6%]; 95% CI, 0.3%-2.5%; P = .02) compared with placebo at 18 months, with the greatest differential increase observed in the basal region (overall least-squares mean [SE] difference, 2.1% [0.8%]; 95% CI, 0.6%-3.6%; P = .006) and no significant differences in the mid and apical regions among groups | | |  |
| Solomon et al. 2019 | Had a diagnosis of hATTR amyloidosis with a documented TTR mutatiom and symptomatic neuropathy, were ambulatory, had adequate liver function and adequate renal function, and were included in the prespecified cardiac subpopulation (baseline LV wall thickness > 13 mm, no history of aortic valve disease or hypertension)  Enrolled participants which did not fulfill criteria to be included in prespecified cardiac subpopulation | 18 - 85 yo | Patisiran 0.3 mg/kgbb - IV  once / 3 weeks | Cardiac subpopulation  90 | Placebo | 36 | Echo parameters at 18 months  NTproBNP and 10MWT gait speed at 9 months and 18 months | Reduction of left ventricular wall thickness, interventricular septal wall thickness, posterior wall thickness, and relative wall thickness. Increase of end diastolic volume, decrease of global longitudinal strain, increase of cardiac output. Decrease of NT pro BNP. Increase of 10MWT gait speed.  No significant outcome in echo parameters. Decrease of NTproBNP. Increase of 10MWT gait speed. | Reduction in mean LV wall thickness (least-squares mean difference±SEM, –0.9±0.4 mm; P=0.017) was observed with patisiran compared with placebo.  In patisiran treated patients compared with placebo, global longitudinal strain was decreased (–1.4%±0.6%, P=0.015), cardiac output was increased (0.38±0.19 L/min, P=0.044), and LVEDV was increased (8.31±3.91 mL, P=0.036)  Patisiran reduced NT-proBNP compared with placebo at 9 months (ratio of fold change patisiran/placebo, 0.63; 95% CI, 0.50–0.80) and 18 months (ratio of  fold change patisiran/placebo, 0.45; 95% CI, 0.34–  0.59; P=7.7×10–8), corresponding to a 55% reduction  relative to placebo | | | Cardiac serious adverse events  Intervention : 20  Control : 10 |
| Adams et al. 2018 | hereditary transthyretin amyloidosis with polyneuropathy, some patients has cardiac abn (NYHA I dan II) | 18 - 85 years | Patisiran 0.3 mg/kgbb - IV  once / 3 weeks | 148 | Placebo | 77 | 18 months | mNIS+7, LVthickness, LVLStrain | -34; -0.9 ; -1.37  <0.001; 0.02; 0.02 | 28; -0.1; 1.46 | mNIS+7 -6 LVWT (mm) -1 LVLS (%) 0.08 | diarrhea, edema, nausea, cough, asthenia, death, etc  Intervention : 143  Control : 75 |
| Obici et al. 2020 | hereditary transthyretin amyloidosis with polyneuropathy, some patients has cardiac abn (NYHA I dan II) | 18 - 85 years | Patisiran 0.3 mg/kgbb - IV  once/ 3 weeks | 148 (90 with cardiac problems) | Placebo | 77 (36 with cardiac diseases ) | 18 months | measures of overall QoL |  | 14.4 ; 0.6 | Norfolk-6.7; EQ +2 | Not reported |
| REVUSIRAN | | | | | | | | | | | | |
| Judge et al. 2020 | TTR mutation and amyloid deposits, hx of hf and cardiac involvement on echo | 18 - 90 years | Revusiran 500mg/SC | 140 | Placebo | 66 | 18 months | 6MWT, Troponin I, NTpBNP |  | -17.6 | 6MWT -21.4 | the study was prematurely discontinued due to an imbalance of deaths observed in the revusiran group (18 patients, 12.9%) compared with the placebo group (2 patients, 3.0%) during the on-treatment period |
| SLN360 | | | | | | | | | | | | |
| Nissen et al. 2022 | no known CVDs  Lp(a) conc >150 nmol/L  BMI 18-45 kg/m2 | adults  36-63 years | SLN360  SD  30 mg/SC  100 mg/SC  300 mg/SC  600 mg/SC | 24 (6 each dose) | Placebo | 8 | 150 days | Safety and tolerability |  |  |  | Participants with any treatment-emergent adverse event  intervention group : 100%  placebo group : 75% |

**Supplementary material 3: Risk of Bias Assessment (ROBVIS)**

**
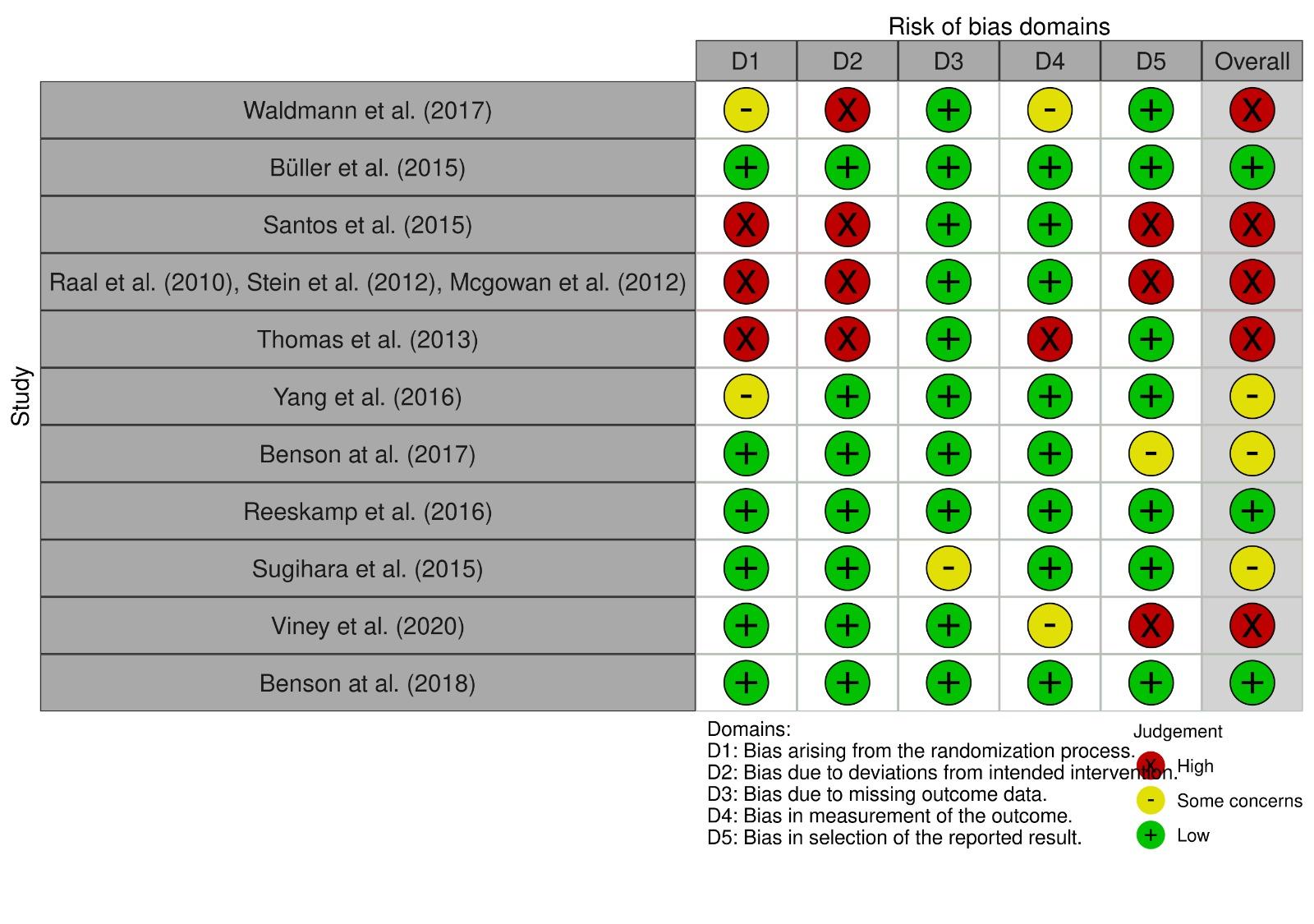
**
